# Enhancement of Efficiency of Perovskite Solar Cells with Hole-Selective Layers of Rationally Designed Thiazolo[5,4-*d*]thiazole Derivatives

**DOI:** 10.1021/acsami.4c04105

**Published:** 2024-05-29

**Authors:** Asta Dabuliene, Zhong-En Shi, Karolis Leitonas, Chien-Yu Lung, Dmytro Volyniuk, Khushdeep Kaur, Vitaly Matulis, Dmitry Lyakhov, Dominik Michels, Chih-Ping Chen, Juozas Vidas Grazulevicius

**Affiliations:** † Department of Polymer Chemistry and Technology, Kaunas University of Technology, Baršausko Str. 59, Kaunas LT-51423, Lithuania; ‡ Department of Materials Engineering and Organic Electronics Research Center, Ming Chi University of Technology, New Taipei City 243, Taiwan; § Belarusian State University, Minsk 220030, Republic of Belarus; ∥ Computer, Electrical and Mathematical Science and Engineering Division, 4700 King Abdullah University of Science and Technology, Thuwal 23955-6900, Saudi Arabia; ⊥ College of Engineering and Center for Sustainability and Energy Technologies, Chang Gung University, Taoyuan City 33302, Taiwan

**Keywords:** solar cell, perovskite, thiazolo[5,4-*d*]thiazole, indoor harvesting, hole-selective materials

## Abstract

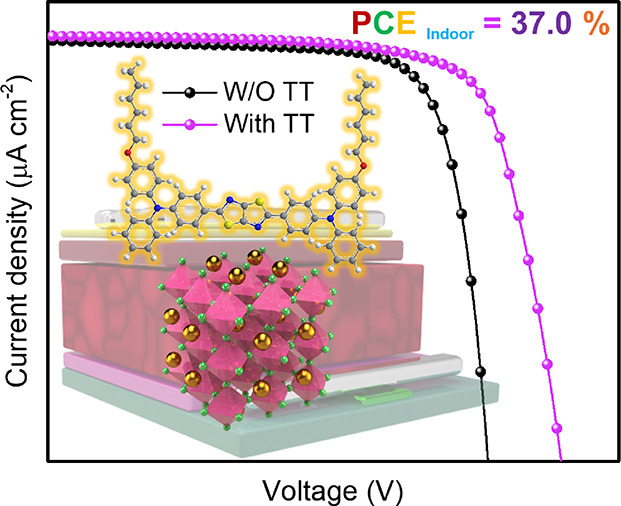

We introduce thiazolo[5,4-*d*]thiazole (TT)-based derivatives featuring carbazole, phenothiazine, or triphenylamine donor units as hole-selective materials to enhance the performance of wide-bandgap perovskite solar cells (PSCs). The optoelectronic properties of the materials underwent thorough evaluation and were substantially fine-tuned through deliberate molecular design. Time-of-flight hole mobility TTs ranged from 4.33 × 10^–5^ to 1.63 × 10^–3^ cm^2^ V^–1^ s^–1^ (at an electric field of 1.6 × 10^5^ V cm^–1^). Their ionization potentials ranged from −4.93 to −5.59 eV. Using density functional theory (DFT) calculations, it has been demonstrated that S0 → S1 transitions in TTs with carbazolyl or *ditert*-butyl-phenothiazinyl substituents are characterized by local excitation (LE). Mixed intramolecular charge transfer (ICT) and LE occurred for compounds containing *ditert*-butyl carbazolyl-, dimethoxy carbazolyl-, or alkoxy-substituted triphenylamino donor moieties. The selected derivatives of TT were used for the preparation of hole-selective layers (HSL) in PSC with the structure of glass/ITO/HSLs/Cs_0.18_FA_0.82_Pb(I_0.8_Br_0.2_)_3_/PEAI/PC_61_BM/BCP/Ag. The alkoxy-substituted triphenylamino containing TT (**TTP-DPA**) has been demonstrated to be an effective material for HSL. Its layer also functioned well as an interlayer, improving the surface of control HSL_2PACz (i.e., reducing the surface energy of 2PACz from 66.9 to 52.4 mN m^–1^), thus enabling precise control over perovskite growth energy level alignment and carrier extraction/transportation at the hole-selecting contact of PSCs. 2PACz/**TTP-DPA-**based devices showed an optimized performance of 19.1 and 37.0% under 1-sun and 3000 K LED (1000 lx) illuminations, respectively. These values represent improvements over those achieved by bare 2PACz-based devices, which attained efficiencies of 17.4 and 32.2%, respectively. These findings highlight the promising potential of TTs for the enhancement of the efficiencies of PSCs.

## Introduction

Perovskite solar cells (PSCs), valued for their high power conversion efficiency (PCE), solution processability, and finely adjustable energy levels, have recently attracted considerable attention.^1−4^ Their versatility is apparent in a variety of applications, from PSCs functioning optimally under one-sun illumination to indoor PSC (iPSCs) capable of harnessing energy from artificial lighting.^5−8^ However, PSCs have yet to play an important role in the photovoltaic market, primarily due to certain limitations related to their relatively low stability in comparison to mature Si–PV devices. In order to improve the performance and stability of PSCs, numerous researchers have focused on interfacial engineering between carrier transport layers (CTLs) and perovskite layers.^9−11^ Charge-selective materials and interfacial layers (IFLs) play crucial roles in the performance of PSCs. They not only accelerate charge extraction but also passivate defect states at interfaces.^12,13^ Recent advancements have highlighted the effectiveness of specificized hole-selective layers (HSL) in inverted-structured (p–i–n) PSCs.^14^ Especially, molecules containing carbazole (Cz), phenothiazine (PTZ), and triphenylamine (TPA) moieties are preferred for their exceptional hole-extraction capabilities.^15−18^ The desired HSL boasts robust thermal stability and superior mobility, along with suitable surface energies. Tailored adjustments are needed when positioning it beneath the perovskite layer to finely regulate perovskite growth, aiming for optimal performance.^19−21^

Thiazolothiazole is known for its planar and rigid structure with extended π-conjugation.^22^ Derivatives with a conjugated thiazolo[5,4-*d*]thiazole (TT) moiety are used in organic field-effect transistors, dye-sensitized solar cells, and PSCs.^23−25^ TT derivatives can be synthesized simply using a Pd-free synthetic route and affordable starting materials.^25,26^ Compounds with Cz, PTZ, and TPA moieties are extensively employed for organic optoelectronic applications.^27−31^ In this work, we designed and synthesized donor–acceptor–donor (D–A–D)-type hole-selective materials, featuring alkoxy- or alkyl-substituted Cz, PTZ, and TPA moieties as donor units and TT as the acceptor moiety. Our design strategy was based on the exploitation of the planar and rigid electron-deficient TT moiety, which can ensure efficient intermolecular π–π overlap and high glass transition temperatures of the derivatives.^22^ Electron-donating carbazolyl, phenothiazinyl, and triphenylamino groups were selected taking into account good charge-transporting properties of the derivatives containing these moieties.^32−34^ Alkoxy groups were attached to the donor moieties taking into account their hydrogen bonding abilities, which result in the improvement of charge-transporting properties.^35^ Meanwhile, attachment of *tert*-butyl groups allows to considerably increase glass transition temperatures (*T*_g_) and morphological as well as thermal stability of molecular glasses.^36^ The synthesized materials exhibit *T*_g_ of up to 187 °C and hole mobility (μ_h_) of up to 1.63 × 10^–3^ cm^2^ V^–1^ s^–1^ at an electric field of 1.6 × 10^5^ V cm^–1^.

The interfaces between the perovskite and the HSL are responsible for most performance losses and interfacial instability in p–i–n PSCs.^37,38^ Modification of the HSL/perovskite interface allows to improve energy level alignment, regulate perovskite crystallization, and reduce interfacial defects.^39,40^ Efficient HSL design entails selecting building blocks with high *T*_g_ and μ_h_ values, as well as suitable ionization potentials and surface energies.^41^ Moreover, these HSLs can serve as defect passivation layers (i.e., IFL) to enhance the performance of PSCs using traditional HTLs such as nickel oxide (NiO_*x*_), [2-(9*H*-carbazol-9-yl)ethyl]phosphonic acid (2PACz), and [2(3,6-dimethoxy-9*H*-carbazol-9-yl)ethyl]phosphonic acid (MeO-2PACz).^42−44^ In this study, we introduce PSCs utilizing 2PACz and TT derivatives as materials for HSL. Additionally, we explore the impact of the layers of TTs as an interlayer to optimize the performance of 2PACz-based PSCs. X-ray diffraction, steady-state and time-resolved photoluminescence (PL) spectrometry, and scanning electron microscopy (SEM) were utilized to assess the phenomena of performance alteration. Transient photovoltage decays (TPV) and photocurrent decays (TPC) were analyzed alongside the results of charge carrier mobility measurements to evaluate carrier transport and extraction within the devices. Consequently, the optimized wide-bandgap Cs_0.18_FA_0.82_Pb(I_0.8_Br_0.2_)_3_ PSC exhibited a PCE of 19.1% under one-sun (AM 1.5G, 100 mW cm^–2^) illumination, while indoor PSCs reached maximum PCEs of 37.0% under 3000 K LED illumination of 1000 lx. These findings disclose the potential of D–A–D-type molecule-based interface modification as a practical and efficient approach to enhance passivation and contact properties, thereby advancing the development of high-efficiency PSCs.

## Results and Discussion

### Synthesis

**Scheme 1:**
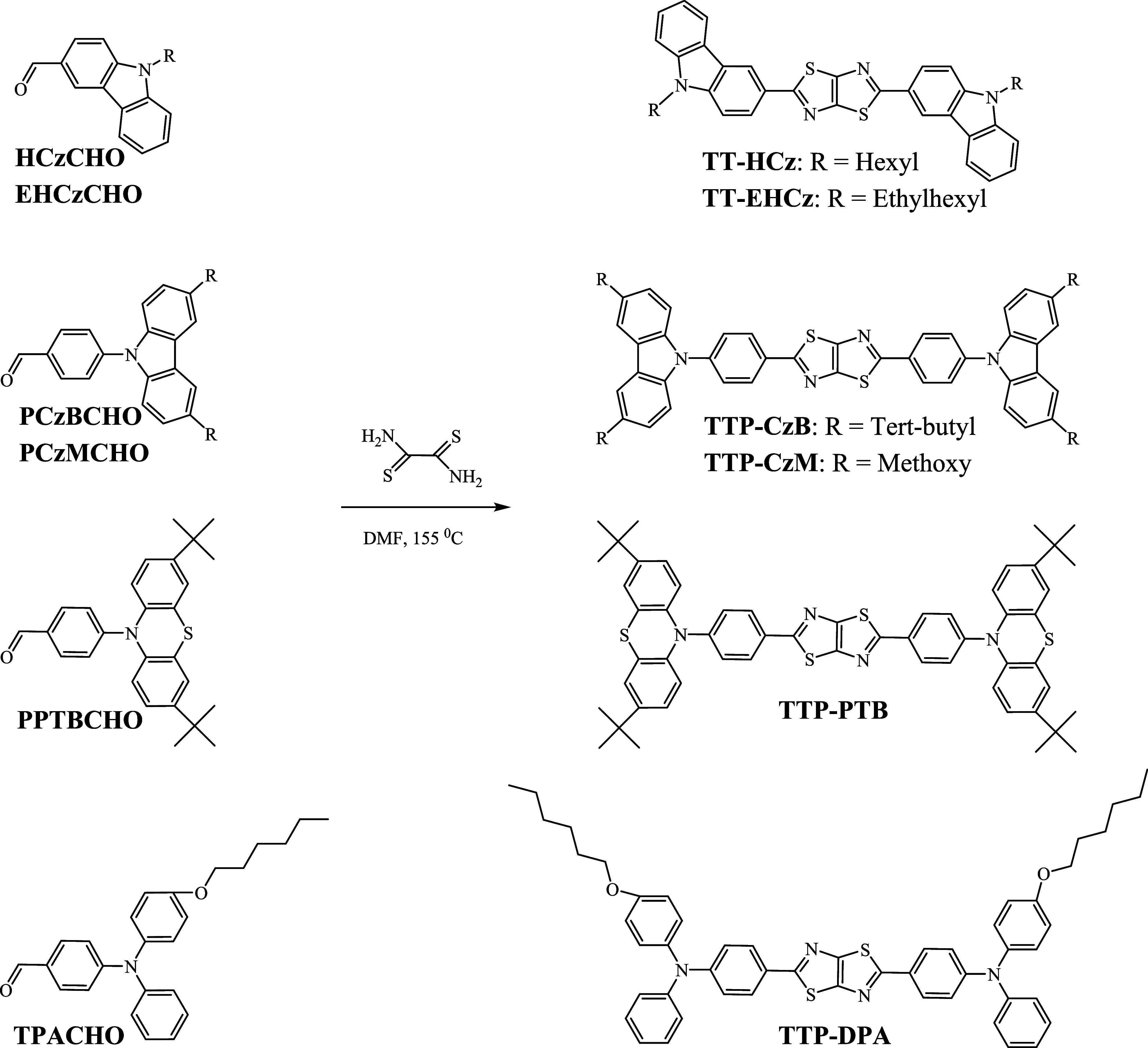
Synthesis of TT Derivatives

Donor-disubstituted TT derivatives were synthesized as shown in Scheme 1. TT-based compounds were obtained from the respective monoformyl derivatives and dithiooxamide in dry dimethylformamide at the reflux temperatures. The products were purified by column chromatography. All the synthesized compounds were identified by mass spectrometry, ^1^H NMR spectroscopy (Figures S1–S4), and ^13^C NMR spectroscopy. The data were found to be in good agreement with the proposed structures.

### DSC AND TGA Studies

**Figure 1:**
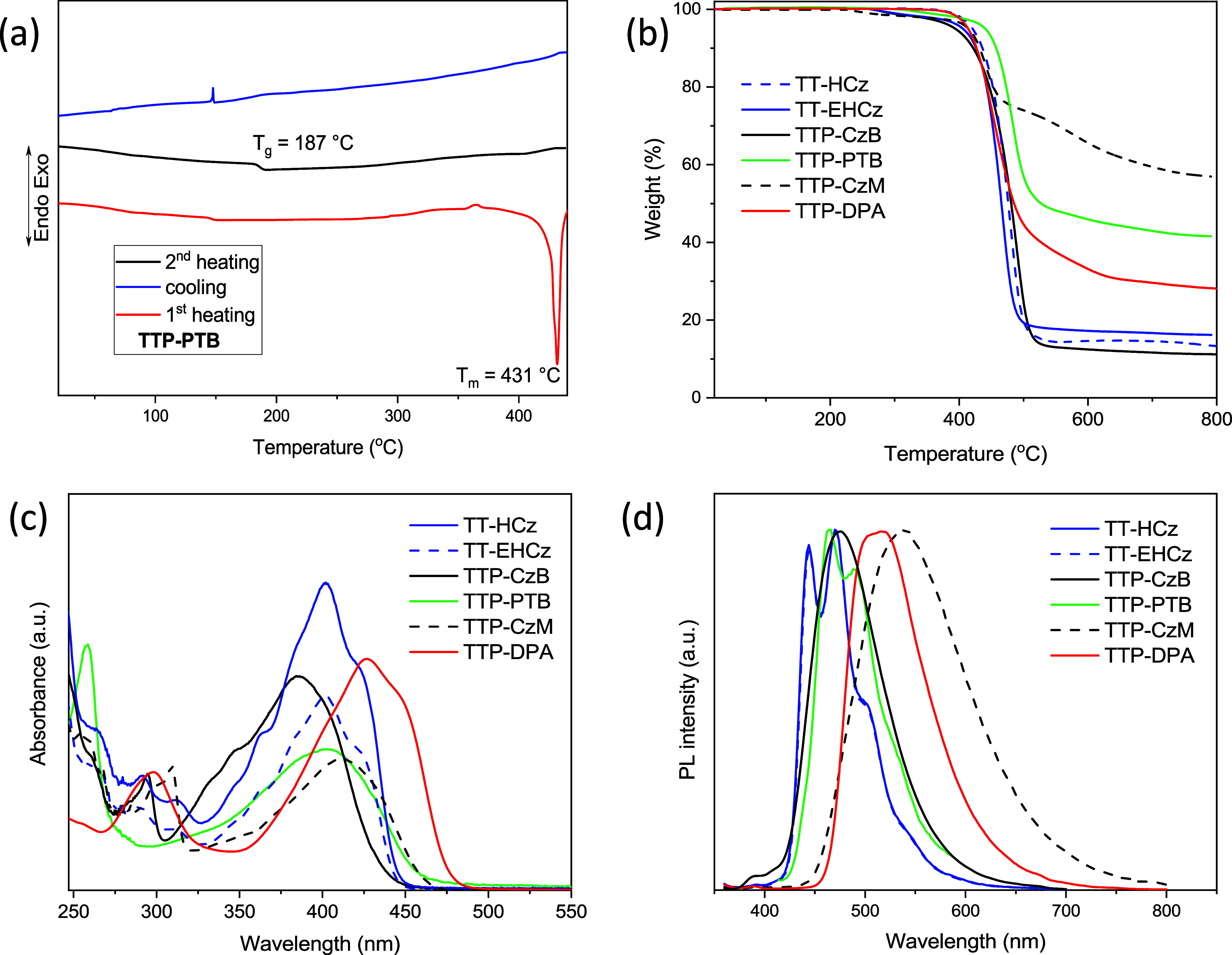
DSC curves of compound **TTP-PTB** recorded at the heating rate of 10 °C min^–1^ in a nitrogen atmosphere (a). TGA curves of TT-based compounds recorded at the heating rate of 20 °C min^–1^ in a nitrogen atmosphere (b). Absorption (c) and PL (d) spectra of the dilute THF solutions of TT derivatives.

The behavior under heating of the synthesized TT derivatives was studied by thermogravimetric analysis (TGA) and differential scanning calorimetry (DSC) under a nitrogen atmosphere. The TGA curves of the compounds are shown in Figure 1b. The results obtained are collected in Table 1. The TT derivatives demonstrated high thermal stability. Their 5% weight loss temperatures were found to be higher than 390 °C. They ranged from 397 to 439 °C. The substituents and their position did not significantly affect the temperature of the onset of weight loss. TGA curves of all the synthesized compounds showed nonvolatile residues at 800 °C with the percentage exceeding 14%. The considerable amounts of nonvolatile residues were also observed for the earlier reported derivatives of TT.^25,45^ This observation shows that under heating, the samples of TT-based compounds are subjected to thermal decomposition but not to sublimation. The relatively high amounts of nonvolatile residues can be explained either by incomplete decomposition of the compounds or by cross-linking of the molecules. Most of the synthesized TT derivatives except TTP-CzM containing methoxy groups exhibited single-stage thermal degradations. TT-based compounds were isolated as crystalline substances after their synthesis (Figure S5, **1a**). Compound **TT-EHCz** containing branched alkyl chains at the nitrogen atoms of Cz moieties showed glass transition at 46 °C in the second DSC heating scan. However, this sample was not morphologically stable and was recrystallized at 141 °C during the same DSC heating scan. A similar behavior was observed for compound **TTP-CzB**. **TTP-CzB** with *tert*-butyl-disubstituted Cz donor units formed molecular glass with high *T*_g_ of 130 °C. However, this sample was also not morphologically stable and showed crystallization signal in the same DSC heating scan. Compound **TTP-CzM** containing methoxy-disubstituted Cz donor moieties did not form molecular glass. Its sample showed an endothermic melting signal at 298 °C in the first DSC heating scan. In the cooling scan, the melted sample showed the exothermic crystallization signal at 227 °C. The following heating scan revealed only melting peaks again. The presence of double melting peaks at 282, 297 °C and 270, 298 °C in the DSC curves of **TTP-CzB** and **TTP-CzM**, respectively, shows that these compounds exhibited polymorphism. The DSC results showed that addition of the phenyl group to the molecule increased glass transition and melting temperatures. DSC measurements showed that compounds **TTP-PTB** and **TTP-DPA** formed relatively morphologically stable molecular glasses. The DSC measurements revealed melting peaks only in the first DSC heating scans of the compounds. Neither melting nor crystallization was observed either in the second or in the following DSC scans of the compounds. Compound **TTP-PTB** containing *tert-*butyl-disubstituted PTZ donor moieties showed very high *T*_g_ of 187 °C (Figure 1a), while compound **TTP-DPA** containing triphenylamino moieties showed glass transition at 46 °C.

**Table 1:** Thermal, Electrochemical, and Photoelectrical Characteristics of TT Derivatives

	**TT-HCz**	**TT-EHCz**	**TTP-CzB**	**TTP-PTB**	**TTP-CzM**	**TTP-DPA**
*T*_–5%_^a^ [°C]	419	405	397	439	412	414
*T*_g_^b^ [°C]		46	130	187		46
*T*_m_^c^ [°C]	222	164	293	431	298	133
*T*_cr_^d^ [°C]	144	141	201		227	
*E*_ox_ vs Fc [V]^e^	0.27	0.16	0.40	–0.21	–0.29	0.08
*E*_red_ vs Fc [V]	–1.84	–1.63	–1.63	–2.29	–2.28	–2.47
IP_CV_ [eV]	5.07	4.96	5.20	4.59	4.51	4.72
EA_CV_ [eV]	3.23	3.40	3.57	2.30	2.23	2.25
*E*_g_^opt^	2.65	2.77	2.82	2.68	2.54	2.59
IP_EP_ [eV]	5.06	4.93	5.67	5.47	5.35	5.01
μ_0_ [cm^2^/V·s]	3.79 × 10^–8^	7.61 × 10^–8^	1.3 × 10^–4^	3.59 × 10^–7^	5.08 × 10^–5^	*h*:6.16 × 10^–5^*e*:1.34 × 10^–4^
μ_h_ [cm^2^/V·s]	4.33 × 10^–5^^f^	7.03 × 10^–5^^f^	3.11 × 10^–4^^f^	4.10 × 10^–4^^f^	1.63 × 10^–3^^g^	*h*:3.97 × 10^–4^^g^*e*:6.69 × 10^–4^^g^
λ_h_ [meV]	397.0	398.3	160.7	502.6	136.7	321.0

a *T*_–5%_—5% mass loss temperature determined by TGA, heating rate 20 °C/min, N_2_ atmosphere.

b *T*_g_—glass transition temperature, *T*_m_—melting point, *T*_cr_—crystallization temperature, determined by DSC, scan rate 10 °C/min, N_2_ atmosphere: second heating scan.

c *T*_g_—glass transition temperature, *T*_m_—melting point, *T*_cr_—crystallization temperature, determined by DSC, scan rate 10 °C/min, N_2_ atmosphere: first heating scan.

d *T*_g_—glass transition temperature, *T*_m_—melting point, *T*_cr_—crystallization temperature, determined by DSC, scan rate 10 °C/min, N_2_ atmosphere: cooling.

e *E*_ox_, *E*_red_—onset potentials of oxidation and redaction, respectively. Ionization potentials (IP_CV_) and electron affinities (EA_CV_) estimated by CV according to the equations IP_CV_ = 4.8 + *E*_ox_ vs Fc and EA_CV_ = IP_CV_ – *E*_red_vsFc. Ionization potential (IP_*e*p_) measured by PE spectrometry. λ_h_—theoretically calculated values.

f Hole drift mobility (μ) estimated by the TOF technique at electric field of 4.9 × 10^5^ V/cm.

g Hole drift mobility (μ) estimated by the TOF technique at electric field of 1.6 × 10^5^ V/cm.

### Photophysical Properties

UV–vis absorption spectra of the dilute solutions of TT derivatives are shown in Figure 1c. The synthesized compounds absorbed electromagnetic radiation in the range of up to 477 nm. The low energy band at 386 nm with the shoulder at ca. 406 nm was observed for compound **TTP-CzB**. This band is very similar to the low energy band of TT and its derivatives.^22^ This observation allows to suppose that the low energy band of compound **TTP-CzB** is mainly caused by locally excited (LE) states of thiazolo[5,4-*d*]thiazole. This is in agreement with the data of the theoretical calculations (see Theoretical Calculations), according to which only a weak intramolecular charge transfer (ICT) is observed for compound **TTP-CzB**. Replacement of a Cz fragment with the triphenylamino moiety in the TT derivatives as well as replacement of *tert*-butyl groups with methoxy groups led to the bathochromic shift of the lowest energy absorption bands by more than 20 nm. The shapes of the absorption bands of TT-based compounds are similar. The observed bathochromic shifts of the bands of compounds **TTP-CzB**, **TTP-PTB**, **TTP-CzM**, and **TTP-DPA** with respect to those of compounds **TT-HCz** and **TT-EHCz** are apparently caused by the enhanced conjugation due to the stronger donating abilities of the donor units. However, the effect of ICT between electron-accepting and electron-donating moieties on the shape and position of absorption spectra of compounds **TTP-CzB**, **TTP-CzM**, and **TTP-DPA** should also be taken into account. This is supported by the red shifts of emission spectra of the solutions of TT derivatives in the solvents with increasing polarities (Figure S6) and the data of theoretical calculations (see Theoretical Calculations). The normalized PL spectra of the dilute THF solutions of the TT derivatives are shown in Figure 1d. The PL spectrum of compound **TTP-DPA** containing triphenylamino groups exhibited red shifts by more than 40 nm compared to the spectra of the carbazolyl-containing compounds **TT-HCz**, **TT-EHCz**, and **TTP-CzB** and the PTZ-containing compound **TTP-PTB**. The PL spectrum of compound **TTP-CzM** containing methoxy-carbazolyl fragments was found to be the most red-shifted. This can be explained by the fact that the excitation of compound **TTP-CzM** is accompanied by the most efficient charge transfer (see Theoretical Calculations). The vibrational PL spectra of the solutions of **TT-HCz**, **TT-EHCz**, and **TTP-PTB** in THF can apparently be attributed to the local excitation of the molecules, while structureless PL spectra of the solutions of **TTP-CzB**, **TTP-CzM**, and **TTP-DPA** in THF indicate ICT's nature. This is confirmed by the results of the theoretical calculations (see Theoretical Calculations for details). According to the calculations, the ICT nature of excited states of compounds **TTP-CzB, TTP-CzM**, and **TTP-DPA** can be explained by the fact that the 2,5-diphenylthiazolo[5,4-*d*]thiazole core and diphenylamine or Cz units are twisted. This effect was not observed in the case of compounds **TT-HCz**, **TT-EHCz**, and **TTP-PTB**, in which the 2,5-dicarbazol-3-yl-thiazolo[5,4-*d*]thiazole core or 2,5-diphenylthiazolo[5,4-*d*]thiazole unit is planar.

### Electrochemical and Photoelectrical Properties

Cyclic voltammetry (CV) measurements were performed to study electrochemical properties of TT-based compounds. All the compounds showed reversible oxidation after repeated scans (Figure S7, **2a**). The shapes of the CV curves of TT derivatives remained unchanged after several cycles. This observation shows formation of both stable radical cations and radical anions.

**Figure 2:**
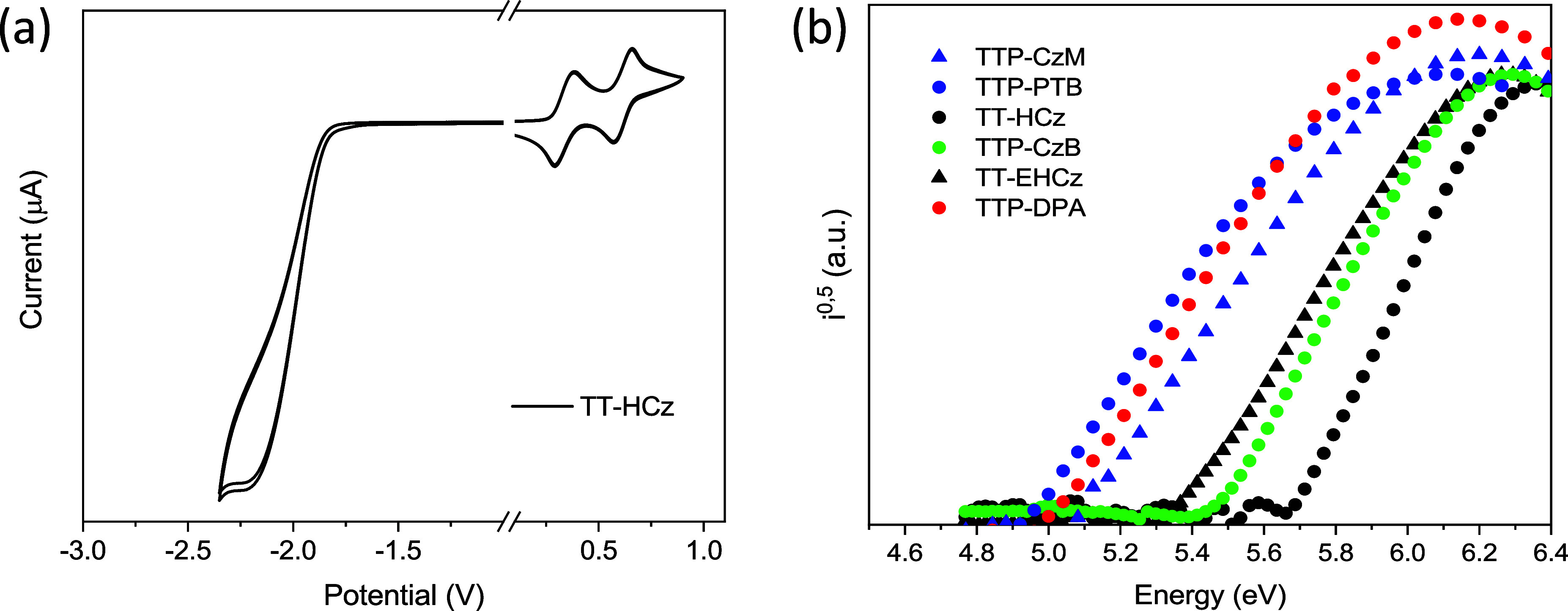
Cyclic voltammetry of compound **TT-HCz** recorded for 0.1 M Bu_4_NBF_4_/dichloromethane solution at a scan rate of 0.1 mV s^–1^ (a) and photoelectron emission spectra of vacuum-deposited films of TT derivatives (b).

Ionization potential (IP_CV_) values were estimated from oxidation onset potentials against ferrocene (*E*_ox_ onset versus Fc). The IP_CV_ values of the TT derivatives ranged from 4.51 to 5.20 eV (Table 1). The replacement of the Cz fragment by the PTZ moiety resulted in the decrease of the IP_CV_ value by 0.61 eV (cf. IP_CV_ of **TTP-CzB** and **TTP-PTB**). The replacement of *tert-*butyl groups by methoxy groups also resulted in the decrease of the IP_CV_ value by 0.69 eV (cf. IP_CV_ of **TTP-CzB** and **TTP-CzM**). The electron affinity (EA_CV_) values of TT compounds were found to fall in the range of 2.23–3.57 eV. The EA_CV_ values were determined from the values of IP_CV_ and reduction potential onset with respect to Fc. The ionization potentials (IP_EP_) of the solid layers of the compounds were established by the photoelectron emission method in air (Figure 2b). The values ranged from 4.93 to 5.67 eV (Table 1). Both the methods of estimation of ionization potentials demonstrated similar patterns. Higher IP_EP_ values relative to the IP_CV_ values were obtained since more energy was needed to knock out electrons from the compounds in the solid state than in solutions.^46^

### Charge-Transporting Properties

**Figure 3:**
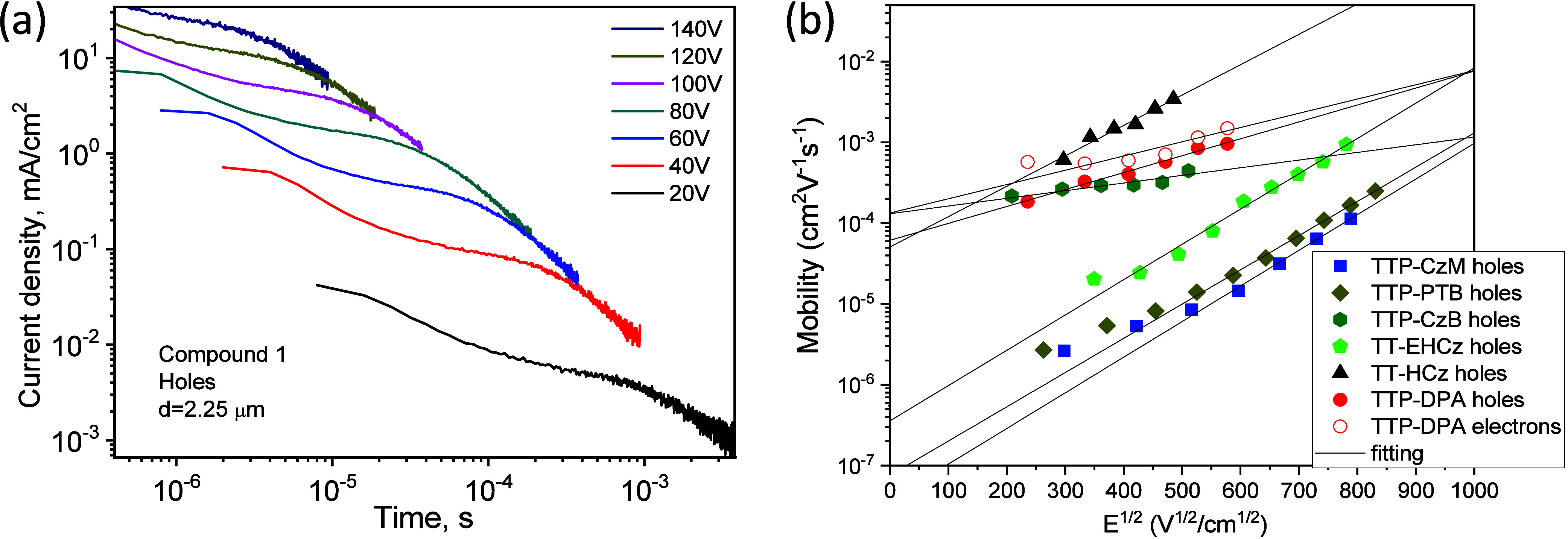
TOF signals for holes in the film of compound **TT-HCz** (a) and electric field dependences of hole mobilities (b) for the layers of TT derivatives and of electron mobilities for the layer of compound **TTP-DPA**.

Charge-transporting properties of the layers of the synthesized derivatives of TT were estimated by the time-of-flight (TOF) technique. TOF signals were recorded applying the positive and negative voltages at the ITO electrode, thus detecting transport of holes or electrons (Figure 3a and Figure S8). The hole transport was detected for all the studied compounds, while the electron transport was proved only for compound **TTP-DPA**. The charge transport in the layers of the TT derivative was found to be dispersive. The experimental mobility values are in good agreement with the Poole–Frenkel relation μ = μ_0_e^β*E*^1/2^^, where μ_0_ is the zero-field mobilities and β is the field dependence parameter. The values of μ_0_ for the layers of TT-based compounds were obtained in the range from 3.79 × 10^–8^ to 1.34 × 10^–4^ cm^2^/V·s. At a high electric field of 1.6 × 10^5^ V/cm, the highest hole mobility value of 1.63 × 10^–3^ cm^2^/V·s was observed for compound **TTP-CzM** (Table 1). Both hole and electron transports were detected for the layer of **TTP-DPA** (Figure 3b). The other compounds exhibit a unipolar behavior, i.e., hole transport with the measurable mobility for holes only (Figure 3b). 9-*N*-Substituted Cz derivatives (**TTP-CzB**, **TTP-CzM**) showed considerably higher hole mobilities than the 3*H*-substituted Cz compounds (**TT-HCz, TT-EHCz**). The replacement of the PTZ fragment by the Cz fragment as well as the *tert*-butyl groups by methoxy groups resulted in the increase in the hole mobilities (Table 1). One of the key parameters defining the charge-transfer rate constant is charge reorganization energy (λ), which is divided into the external (λ_ext_) and internal (λ_int_) parts. λ_ext_ is not considered in this work because of the complexity of the calculation. However, for compounds containing π-conjugated systems, λ_int_ considerably exceeds λ_ext_.^47,48^ Therefore, λ_int_ is a good index for estimation of charge drift mobility. λ_int_ for the hole transfer (λ_h_) was calculated according to eq 1(48) in the gas phase within density functional theory (see Theoretical Calculations below):
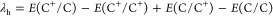
1where *E*(C^+^/C) represents the total energy of single-point C^+^ cations having the geometry of C neutral molecule and *E*(C^+^/C^+^) represents the total energy of C^+^ ions having optimized geometry.

The values of λ_h_ calculated for the studied compounds are presented in Table 1. The changes of λ_h_ values for the series of the compounds are consistent with the changes of the experimental μ_h_ values, with the exception of compound **TTP-PTB** (Table 1). Thus, the lowest hole mobility was observed for compounds **TT-HCz** and **TT-EHCz**. These compounds are also characterized by the highest values of internal reorganization energies, which can be attributed by the larger and, therefore, more flexible quasi-planar π-conjugated system (**TT** with two Cz moieties vs **TT** with two phenyl moieties in the case of the other compounds, see Figure S9). Compounds **TTP-CzB** and **TTP-CzM** are characterized by the lowest values of internal reorganization energies, since they have two types of rigid π-conjugated systems, i.e., **TTP** and **Cz**. Due to the rigidity, the geometries of cations and neutral molecules of **TTP-CzB** and **TTP-CzM** are similar. Therefore, the calculated λ_h_ values are small. The large value of λ_h_ observed for compound **TTP-PTB** is explained by the different geometries of neutral molecule and cation, caused by the increase in the angle between **TTP** and **PTB** moieties in cation. Presumably, such unfolding of the structure, in the case when the molecules form layers, is difficult. Therefore, the calculated value of the internal reorganization energy for compound **TTP-PTB** does not agree with the experimental μ_h_.

### Theoretical Calculations

**Figure 4:**
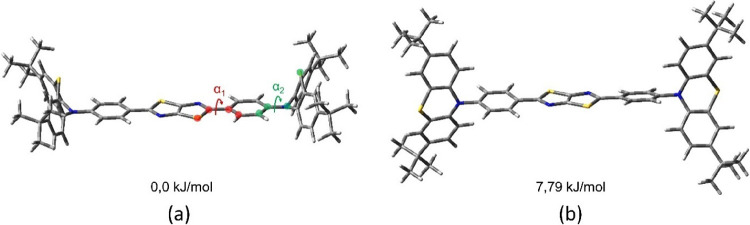
MN15/6-31+G(d) optimized in THF geometries of quasi-axial (a) and quasi-equatorial (b) conformers of compound **TTP-PTB** and their relative energies.

The geometry and electronic structures of the compounds in ground and excited states were calculated within density functional theory (DFT) using the MN15^49^ functional with the 6-31+G(d) basis set in solution of THF. Solvation effects were considered using the SMD model^50^ in terms of the linear response scheme.^51^ For the calculations of the absorption spectra, the geometries of the ground states (S0) of the compounds were fully optimized in solution with subsequent calculation of the spectra within the TD-DFT approach. The vibration frequencies were calculated for all the optimized structures to confirm that they correspond to the minima of the potential energy surfaces. For the calculations of the PL spectra, the geometry of the first singlet excited state (S1) was fully optimized in solution within TD-DFT. All the optimized structures in the ground (S0) and excited (S1) states had a point group of C_2_. In the case of compound **TTP-PTB**, containing PTZ moieties, we have considered both quasi-axial and quasi-equatorial conformers (Figure 4). The results of our calculations show that the quasi-axial conformer has almost 8 kJ/mol lower total energy value than the quasi-equatorial one in the ground state (Figure 4) and almost 9 kJ/mol lower energy in the first excited state. This corresponds to the molar fraction of the quasi-equatorial conformer of less than 5% in the equilibrium mixture at a temperature of 298 K. Moreover, the value calculated for the quasi-equatorial conformer of the absorption maximum (λ_ABS_(calc.) = 367 hm) is underestimated compared to the experimental value (λ_ABS_(exp.) = 403 nm). The calculated values of |HOMO and LUMO energies (−6.05 and −1.77 eV) do not match the experimentally determined changes in IP_CV_ and EA_CV_ in a row of considered compounds. Therefore, further in this paper, the results of calculations will be analyzed only for the quasi-axial conformer of compound **TTP-PTB**. All the calculations were carried out using the Gaussian 16 program.^52^ The analysis of electron density was performed using Multiwfn software.^53^

**Figure 5:**
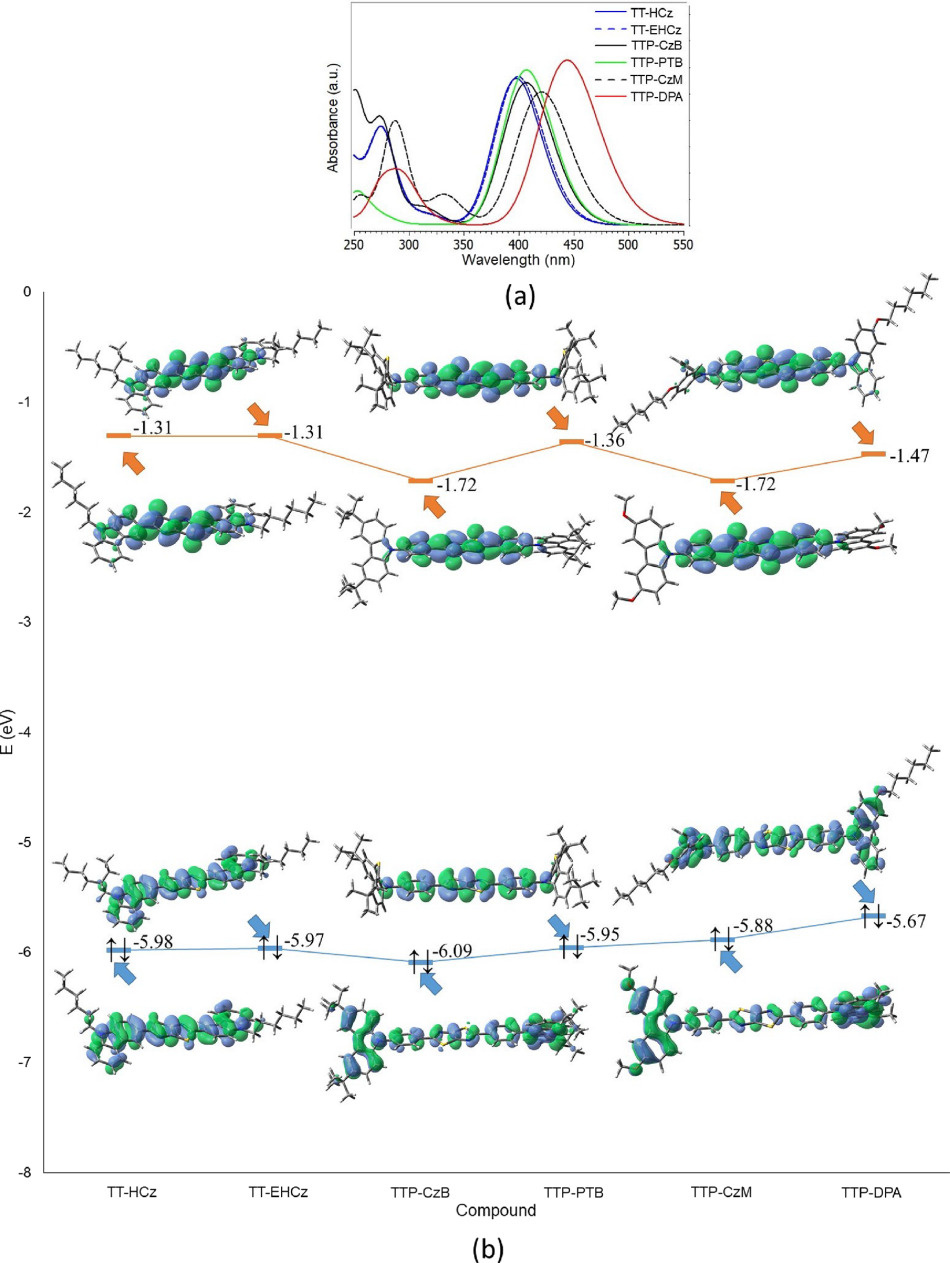
MN15/6-31+G(d) calculated absorption spectra (a). Shapes and energies of HOMO and LUMO (b) of TT-based compounds in THF.

**Figure 6:**
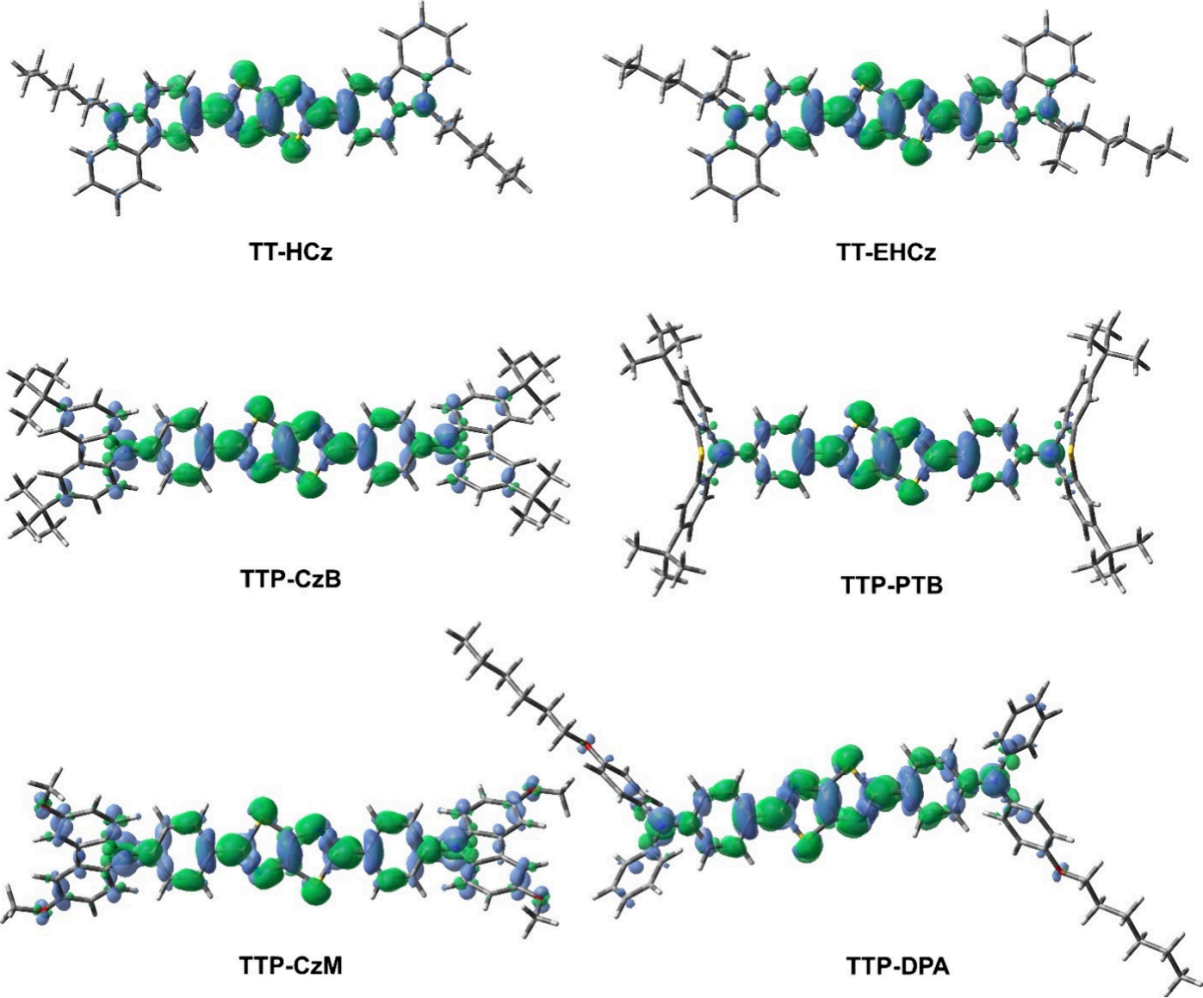
MN15/6-31+G(d) calculated for TT-based compounds in THF plots of Δρ S1–S0. Green (blue) regions indicate increase (decrease) in ρ upon electronic transition.

The calculated wavelengths corresponding to the first absorption maxima (λ_ABS_^1^), wavelengths corresponding to the transition S1 → S0 in the PL spectra (λ_PL_), dihedral angles between TT and Cz or phenyl units (α_1_, see Figure 4) and dihedral angles between phenyl and Cz, PTZ, or diphenylamine units (α_2_, see Figure 4), values of the largest coefficients in the CI expansion (c), oscillator strengths (*f*), energy gap between the singlet and triplet states (Δ*E*_S-T_) and overlaps between functions C_+_ and C_–_ (*S*_+–_ -indexes^53^ are given in Table 2. The calculated absorption spectra of considered compounds as well as the shapes and energies of HOMO and LUMO are presented in Figure 5. The calculated plots of Δρ S1–S0 are given in Figure 6. The shapes of natural transition orbitals (NTOs) for S1 → S0 transitions are presented in Figures S10 and S11 of the Supporting Information.

**Table 2:** MN15/6-31+G(d) Calculated for TT-Based Compounds in THF, the Wavelengths Corresponding to the First Absorption Maxima (λ_ABS_^1^), Wavelengths Corresponding to Maxima of PL Spectra (λ_PL_), Dihedral Angles between Donor and Acceptor Moieties (α), Values of the Largest Coefficients in the CI Expansion (*c*), Oscillator Strengths (*f*), Overlaps between Functions C_+_ and C_–_ (*S*_+–_ -Index) and Energy Gap between the Singlet and Triplet States (Δ*E*_S-T_)

parameter	**TT-HCz**	**TT-EHCz**	**TTP-CzB**	**TTP-PTB**	**TTP-CzM**	**TTP-DPA**
λ_ABS_^1^ [nm]	398 (402)^a^	399 (401)	407 (386)	407 (403)	421 (413)	444 (427)
α_1_ and α_2_ [°]	13	11	5 and 52	0 and 90	2 and 52	6 and 30
*c*(HOMO→LUMO)	0.677	0.678	0.624	0.681	0.622	0.662
*f*	1.995	2.022	1.937	2.109	1.812	2.244
*S*_+–_ -index	0.930	0.952	0.922	0.961	0.907	0.928
Δ*E*_S-T_ [eV]^b^	0.765	0.765	0.757	0.797	0.684	0.649
λ_PL_ [nm]	507 (444, 470)	508 (444, 470)	512 (476)	523 (464, 488)	520 (536)	550 (516)
α_1_ and α_2_ [°]	0	1	0 and 46	1 and 90	1 and 46	0 and 34
*c*(HOMO→LUMO)	0.693	0.693	0.673	0.691	0.657	0.681

a The experimental values of λ_ABS_^1^ and λ_PL_ are given in brackets.

b Energy gap between the singlet and triplet states (Δ*E*_S-T_) was calculated as energy difference between S1 and T1 excited states having the ground state geometry in THF.

The calculated spectra within MN15/6-31+G(d) method absorption of TT-based compounds (Figure 5) are in excellent agreement with the experimental ones (Figure 1c). The shapes of the absorption bands of synthesized TT compounds are similar. The lowest energy band maxima lie in the range of 390–450 nm (Table 2), which is in good agreement with the experimental wavelengths of absorption maxima. The largest difference between the calculated and experimental values of λ_ABS_^1^ is observed for compound **TTP-CzB**, but it is only of 21 nm. Compound **TTP-CzB** is characterized by the broad first absorption band (see Figure 1c).

According to the calculations for all the studied compounds, the first absorption peak in the absorption spectrum can be attributed to HOMO → LUMO transition (see the values of largest coefficients in the CI expansion for S0 → S1 excitation in Table 2). The large values of the calculated *f*, *S*_+–_ -indexes and Δ*E*_S-T_ values show that the first maxima in the absorption spectra of all considered compounds correspond to the mainly local excitation (LE) with the strongest overlap between of S0 and S1 electron densities for **TT-EHCz** and **TTP-PTB** (see the values of S_+–_ -indexes in Table 2). Nevertheless, for compounds **TTP-CzB** and **TTP-DPA** and especially for **TTP-CzM**, the calculated values of *S*_+–_ -indexes are lower than those estimated for **TT-HCz**, **TT-EHCz**, and **TTP-PTB**, which may indicate a weak ICT nature of S0 → S1 transition. Indeed, in the case of compounds **TTP-CzB** and **TTP-CzM**, the significant part of electron density of HOMO is located on Cz moieties (Figure 5), which are turned by 52° with respect to phenyl rings (Table 2). The electron density of LUMO of the compounds is almost completely localized on a planar 2,5-diphenylthiazolo[5,4-*d*]thiazole unit (Figure 5). Therefore, the mixed LE and ICT transition occurs in the case of compounds **TTP-CzB** and **TTP-CzM**. The same situation is observed for compound **TTP-DPA**. As it can be seen from Figure 5, the part of electron density of HOMO is located on the diphenylamino moiety, which is turned by 30° relative to the phenyl ring. Meanwhile, the electron density of LUMO is localized on the planar 2,5-diphenylthiazolo[5,4-*d*]thiazole moiety. In the case of compounds **TT-HCz** and **TT-EHCz**, the first band in the absorption spectra corresponds to the local excitation of the molecules (see the shapes of HOMOs and LUMOs of these compounds in Figure 5) since the TT moiety and Cz units are in the same plane. In compound **TTP-PTB**, the PTZ moieties are turned by 90° relative to the 2,5-diphenylthiazolo[5,4-*d*]thiazole unit. Therefore, the PTZ moieties do not contribute to either HOMO or LUMO (Figure 5). The ICT found for compounds **TTP-CzB**, **TTP-CzM**, and **TTP-DPA** can be seen more clearly in Figure 6. For these structures, especially in the case of compound **TTP-CzM** containing donor methoxy groups, the blue region of electron density is concentrated on the outermost moieties (Cz or diphenylamine).

The changes of HOMO energies for the series of TT-based compounds (Figure 5) are consistent with the changes of the experimental IP_CV_ values (Table 1). Thus, compounds **TTP-PTB**, **TTP-CzM**, and **TTP-DPA** have slightly higher HOMO energies and lower IP_CV_ values with respect to those of compounds **TT-HCz**, **TT-EHCz**, and **TTP-CzB**. Compound **TTP-CzB** is characterized by the lowest HOMO and the highest IP_CV_ values. It should be noted that the changes in HOMO energies in the series of considered compounds are not as pronounced as the changes in the IP_CV_, and the absolute values of the HOMO energies do not coincide with the values of the IP_CV_. This is not surprising, since the ionization leads to the change of the distribution of the total electron density in a molecule. The changes of LUMO energies for the series of TT-based compounds (Figure 5) are less consistent with the changes of EA_CV_ values (Table 1). This observation is explained by the difficulties of accurately calculating the energies of virtual orbitals within DFT.

The calculated wavelengths of maxima of the PL spectra of TT derivatives are in worse agreement with the experimental values than those of the absorption spectra (Table 2). This observation is explained both by the difficulty of optimization of the geometry for the excited state and by the higher error when considering the influence of the solvent in the framework of the LR scheme. The geometries of the compounds in the S1 excited state and the nature of S1 → S0 transitions are similar to those observed for the ground state. The analysis of MN15/6-31+G(d) calculated plots of S1 → S0 NTOs shows that the PL spectra of compounds **TT-HCz**, **TT-EHCz**, and **TTP-PTB** can be attributed to the local excitation of the planar 2,5-dicarbazol-3-yl-thiazolo[5,4-*d*]thiazole moiety or 2,5-diphenylthiazolo[5,4-*d*]thiazole unit (Figure S10). PL spectra of compounds **TTP-CzB**, **TTP-CzM**, and **TTP-DPA** the PL correspond to the mixed LE-ICT transitions, in which the charge transfer from twisted Cz or diphenylamino units to the 2,5-diphenylthiazolo[5,4-*d*]thiazole moiety occurs (Figure S11). The strongest ICT is observed for compound **TTP-CzM**. This explains that the experimental PL spectrum of the compound **TTP-CzM** is the most red-shifted.

### Application in Perovskite Photovoltaics

**Figure 7:**
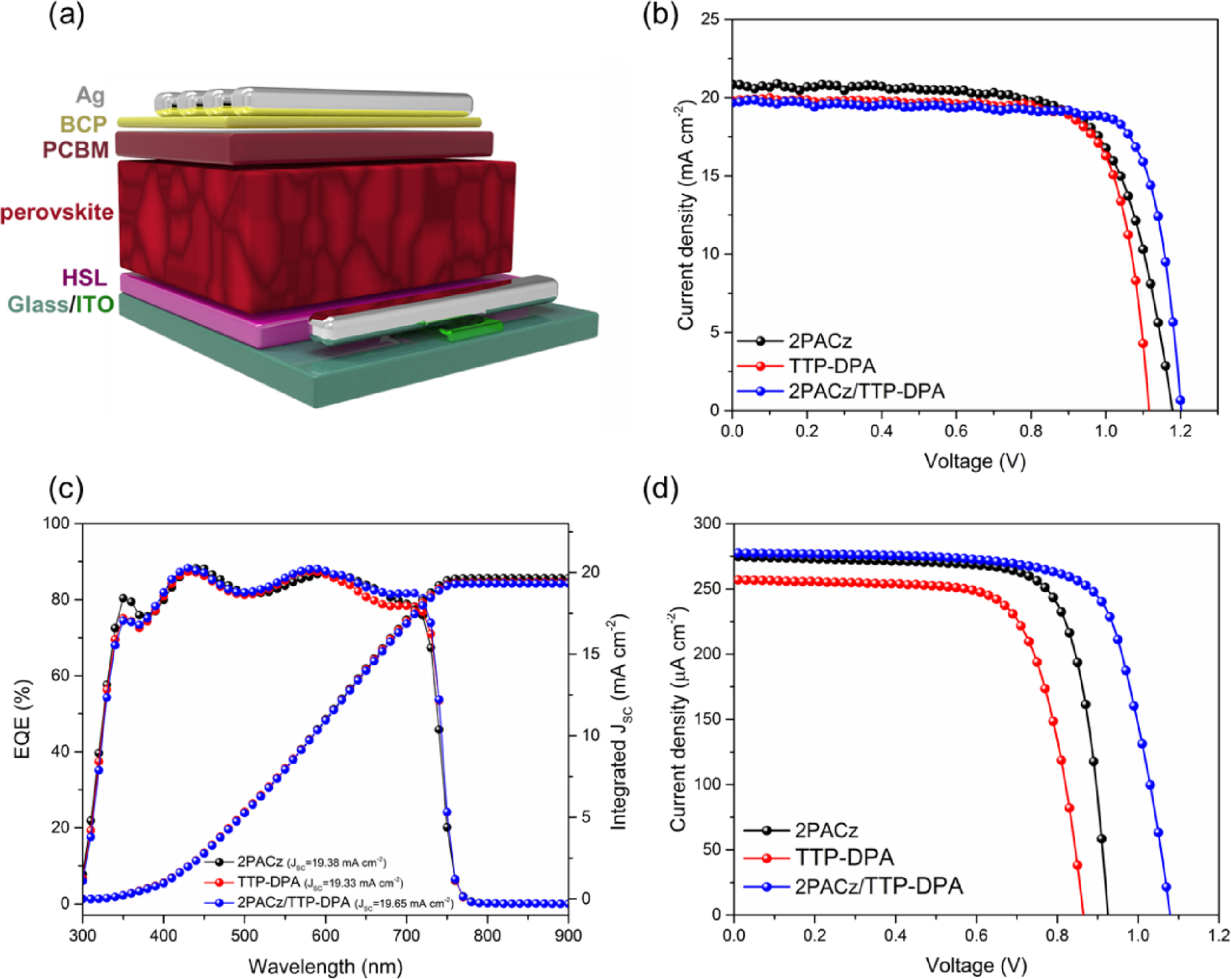
Device architecture (a); *J*–*V* curves from the best PCE results under one-sun illumination (b); EQE characteristic (c) and *J*–*V* curves from the best PCE results under 3000 K LED illumination (d).

To explore the potential of TT-based compounds for hole-selective contacts, we assessed their impact on the performance of PSCs with wide band gaps (WBGs) of 1.70 (Cs_0.18_FA_0.82_Pb(I_0.8_Br_0.2_)_3_) and 1.77 (Cs_0.18_FA_0.82_Pb(I_0.6_Br_0.4_)_3_) eV using an inverted p–i–n device architecture: ITO/HSLs/perovskites/PC_61_BM/BCP/Ag (refer to Figure 7a). We prepared the TT-based compound HSLs on top of ITO using a concentration of 3.0 mg/mL. Further increasing the concentration of the hole-selective materials resulted in poor coverage of the perovskite layer. Detailed fabrication conditions are provided in Experimental Section. Table S2 presents the characteristics of HSL-based Cs_0.18_FA_0.82_Pb(I_0.8_Br_0.2_)_3_ and Cs_0.18_FA_0.82_Pb(I_0.6_Br_0.4_)_3_ PSCs under one-sun illumination (AM 1.5 G 100 mW cm^–2^). Additionally, we prepared HSL-free devices for the comparison. As depicted in Table S2, compound **TTP-DPA** exhibited the best performance under the testing conditions. Notably, the performance of these HSL-containing PSCs is influenced not only by the energy levels, charge mobility, and optoelectronic properties of these HSL compounds but also by factors such as their solubility, film formation, and quality of the films, which determine their suitability for PSCs. Considering the best performance of the **TTP-DPA** device, we focused solely on the study of the Cs_0.18_FA_0.82_Pb(I_0.8_Br_0.2_)_3_ PSCs fabricated using compound **TTP-DPA**. We investigated the effect of the concentration of the solution of **TTP-DPA** on the thickness and quality of the HSL and, consequently, the overall performance of the PSCs. The PCEs of PSCs prepared with the concentrations of **TTP-DPA** of 0.5, 1.0, 1.5, and 3.0 mg/mL were measured to be 7.6, 11.4, 15.1, and 17.1% (Table S2), respectively. The observed trend indicates that an increased thickness of the layer of compound **TTP-DPA** leads to the notable improvement the device performance. The best performance was achieved using the solution with the concentration of 3 mg/mL, with the short-circuit current density (*J*_SC_) of 19.8 mA cm^–2^, an open circuit voltage (*V*_OC_) of 1.09 V, and a fill factor (FF) of 79.3%. To compare the performance of the device with compound **TTP-DPA**, two different PSCs with HSLs of 2PACz (control) and 2PACz/**TTP-DPA** were fabricated and evaluated (Figure 7b and Table S3). The control device with HSL of 2PACz control devices exhibited a PCE of 17.0 ± 0.5% with a *J*_SC_ of 19.4 ± 1.1 mA/cm^2^, a *V*_OC_ of 1.18 ± 0.02 V, and an FF of 74.5 ± 2.4%. These devices exhibited a performance similar to that reported for PSCs having a similar device structure and perovskite composition.^54^ The application of 2PACz continues to face challenges due to suboptimal reproducibility caused by insufficient surface coverage and the existence of voids within the perovskite layer at the interface.^55^ The layer of compound **TTP-DPA** shows great potential to serve as an efficient HSL, compared with that of 2PACz in terms of PCE and FF. This may be attributed to the efficient hole extraction and the decreased number of defect states at the interface. The lower *V*_OC_ of the compound **TTP-DPA**-containing device could be due to a mismatch of energetic alignment. This issue was then addressed by combining 2PACz and **TTP-DPA** to form a bilayer HSL. The device with an HSL of 2PACz/**TTP-DPA** exhibited a PCE of 18.4 ± 0.7% with a *J*_SC_ of 19.6 ± 0.2 mA/cm^2^, a *V*_OC_ of 1.18 ± 0.02 V, and an FF of 79.2 ± 1.5%, outperforming the control device. Figure 7c displays the plots of the external quantum efficiency (EQE) and the integrated *J*_SC_ versus wavelength recorded for the devices, showing the negligible mismatches between EQE response and the *J*_SC_ obtained from *J*–*V* measurement. In addition, we utilized an indoor lighting simulator comprising a 3000 K LED array to evaluate the performance of iPSCs. The corresponding emission spectrum, depicted in Figure S13, aligns with the light harvesting of perovskite Cs_0.18_FA_0.82_Pb(I_0.8_Br_0.2_)_3_. Under illumination from a 3000 K LED source with the intensity of 1000 lx, the average PCE of the optimized device (2PACz/**TTP-DPA**-based) achieved 33.7 ± 2.2%, accompanied by a *J*_SC_ of 262.1 ± 11.4 μA cm^–2^, a *V*_OC_ of 1.00 ± 0.05 V, and an FF of 76.3 ± 2.5%, with the best PCE reaching 37.0% (Figure 7d and Table S3).

**Figure 8:**
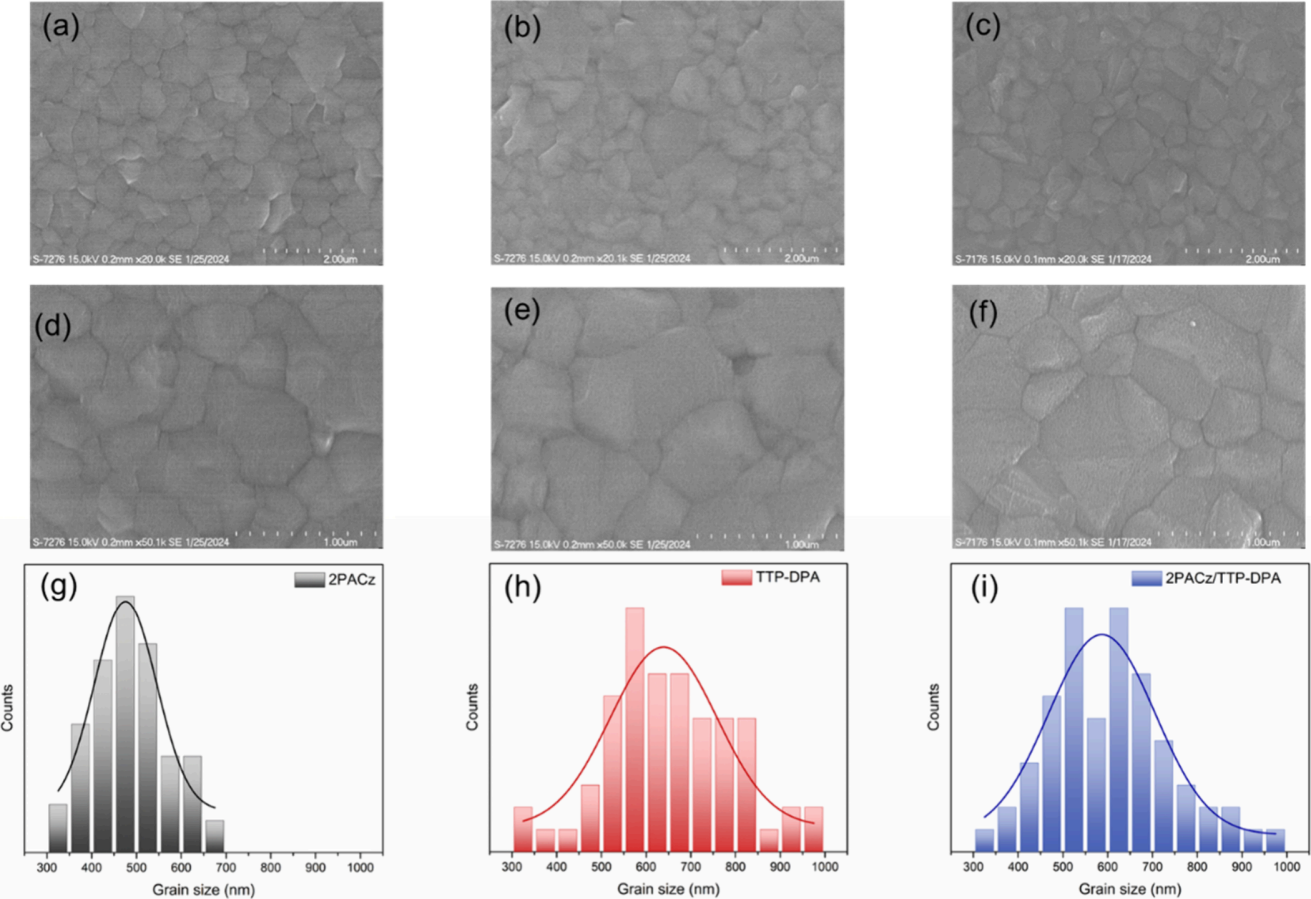
SEM (×20k: a–c) and SEM (×50k: d–f) images of perovskite deposited on 2PACz (a, d), **TTP-DPA** (b, e), and 2PACz/ **TTP-DPA** (c, f). Grain size distribution from SEM images (g–i).

Taking into account the substantial influence of the surface properties on perovskite crystallization, contact angle (CA) analyses were carried out employing water and diiodomethane (DIM) as probe fluids. The results obtained are shown in Figure S14 and in Table S4. The average water contact angles (CA_water_) of 2PACz, **TTP-DPA**, and 2PACz/**TTP-DPA** were found to be of 53.1, 87.9, and 75.2°, respectively, suggesting the increase in hydrophobicity due to the incorporation of compound **TTP-DPA**. Additionally, the surface tension of polar (γ_p_) and dispersive (γ_d_) components along with total surface energy (γ_total_) was calculated from CA_water_ and CA_DIM_ according to the Wu model.^56^ As listed in Table S4, the calculated γ_total_ of 2PACz, **TTP-DPA**, and 2PACz/**TTP-DPA** are of 66.9, 52.4, and 58.2 mN m^–1^, respectively, indicating that compound **TTP-DPA** can mitigate the barrier of high surface energy, significantly influencing perovskite formation.^57^ The crystallization of perovskite was examined using SEM and X-ray diffraction (XRD). All perovskite films exhibited dense and pinhole-free structures (Figure 8a–f) and displayed typical pseudocubic diffraction peaks (Figure S15a). The enlargement of grain size in perovskites deposited on the layers of **TTP-DPA** (Figure 8g–i) indicates the capability of **TTP-DPA** to passivate defects, thereby promoting perovskite growth. As shown in Figure S15a, the diffraction angles (2θ) of 14.1, 20.1, 24.6, and 28.5° correspond to the (1 0 0), (1 1 0), (1 1 1), and (2 0 0) crystal planes of the perovskite, respectively.^58,59^ The distinctive diffraction peak at 12.7° represents residual PbI_2_. However, in the **TTP-DPA-**contacting perovskite film, there is no evident diffraction peak at 12.7°, indicating that the **TTP-DPA** surface fosters perovskite growth, possibly due to its appropriate surface energy in suppressing perovskite grain boundary defects.^60^

We conducted PL measurements using the sample structure of ITO/HSL/perovskite to investigate the influence of compound **TTP-DPA** on the optoelectronic properties. As illustrated in Figure S15b, the trend of PL intensity is 2PACz/ **TTP-DPA** > 2PACz > **TTP-DPA**. This observation indicates the pronounced nonradiative recombination at the interface between ITO/**TTP-DPA** and perovskite.^55^ When perovskite was deposited on ITO/2PACz and ITO/2PACz/**TTP-DPA** substrates, they exhibited higher PL intensity, implying that 2PACz could significantly alter the energy alignments between ITO and perovskite (also see Table S2). The carrier extraction was studied by time-corrected single photo counting (TCSPC) measurements, as depicted in Figure S15c. **TTP-DPA**-contacting perovskite films exhibited the shorter decay profiles, while 2PACz-contacting perovskite films exhibited the longer carrier lifetime. We attributed this mainly to the suppressed nonradiative recombination at the 2PACz/perovskite interface, which agrees with the stronger PL intensity. Additionally, the ITO/2PACz/**TTP-DPA**/perovskite sample showed the highest PL intensity and a trade-off carrier lifetime, which can be attributed to the presence of **TTP-DPA**, improving the perovskite growth, as evidenced by SEM and XRD results.^40,61,62^

We then conducted TPV and TPC measurements to delve deeper into carrier recombination and extraction dynamics in the devices. TPV analysis aids in assessing the photovoltaic efficiency by evaluating the separation of light-generated charge carriers. The **TTP-DPA**-containing device showed a shorter photovoltage decay lifetime of 2.54 μs (Figure S16a). The 2PACz- and 2PACz/**TTP-DPA**-containing devices showed lifetimes of 4.12 and 3.93 μs, respectively. This indicates a slight mismatch in energy level alignment at the interface, supporting our hypothesis regarding the observed low *V*_OC_ in *J*–*V* measurements. We employed TPC analysis to investigate the collection rate of light-generated charge carriers in optoelectronic devices. The **TTP-DPA**-derived device showed a marginally faster TPC decay lifetime of 0.66 μs (Figure S16b) compared to that of the 2PACz device (0.69 μs), suggesting that **TTP-DPA** performs comparably to the well-established HSL of 2PACz. Additionally, we observed further improvement in carrier extraction when employing the HSL of 2PACz/**TTP-DPA** in the devices. To estimate the average mobility of free charges within devices, the charge extraction of photogenerated charge carriers by linearly increasing voltage (photo-CELIV) was performed. The charge mobility (μ) can be calculated from the equation , where *d* is the thickness of the active layer, *A* is the ramp rate of the applied voltage pulse (using 100 V ms^–1^ in this work), and τ_max_ is the time when the current density reaches the maximum value. The factor (1 + 0.36·Δ*J*/*J*_(0)_) is an empirical correction for the redistribution of the electric field, where *J*_(0)_ is the displacement current offset and Δ*J* is the current overshoot. From the curves shown in Figure S16c, a slightly shorter τ_max_ was obtained for the devices incorporating **TTP-DPA**, giving the higher μ of 1.35 × 10^–3^ and 1.25 × 10^–3^ cm^2^ V^–1^ s^–1^ for PSCs containing the layers of **TTP-DPA** and 2PACz/**TTP-DPA**, respectively. In contrast, the control device with HSL of 2PACz showed μ of 1.18 × 10^–3^ cm^2^ V^–1^ s^–1^. The passivation effect becomes increasingly significant in environments with lower light intensity. Under 1 sun illumination, traps within the perovskite may be filled with many photocarriers. However, in dim light conditions, characterized by low photocarrier density, traps within the perovskite could significantly impact the performance of the PSC. The effect of TTP-DPA on perovskite is dual. On the one hand, it facilitates better perovskite growth; on the other hand, it enhances carrier extraction. Both functions contribute to collecting more photogenerated carriers under low-lighting conditions, consequently improving the *V*_OC_ value.

To gain further insight into the potential of **TTP-DPA** forming highly thermally stable HSL, we conducted the thermal stability tests of the devices without encapsulation using a hot plate at 80 °C. As shown in Figure S16d, after more than 30 h of the thermal treatment, the PCE of the device containing HSL of **TTP-DPA** retained ca. 80% of its initial PCE. In contrast, the PCE of the devices employing 2PACz (i.e., 2PACz- and 2PACz/**TTP-DPA**-based) experienced a rapid decline over the same duration. This remarkable thermal stability underscores the potential of TT derivatives for HSL of the next-generation photovoltaics.^63−65^

## Experimental Section

### Materials

Dithiooxamide was purchased from “Aldrich” and used as received. The solvents were purified and dried using standard procedures.^66^ [2-(9*H*-Carbazol-9-yl)ethyl]phosphonic acid (2PACz, >98%, TCI), cesium chloride (CsCl, 99.9%, ultra dry, Alfa Aesar), formamidinium iodide (FAI, >99.99%, Greatcell Solar), lead iodide (PbI_2_, 99.9985%, Alfa Aesar), lead bromide (PbBr_2_, 99.99%, Alfa Aesar), ethanol (anhydrous, ECHO), bathocuproine (BCP, Aldrich), dimethyl sulfoxide (DMSO, Sigma-Aldrich), *N*,*N*-dimethylformamide (DMF, Acros Organics), phenethylammonium iodide (PEAI, Greatcell Solar), and PC_61_BM (Nano-C) were used as received. The starting and some target compounds were synthesized by the reported procedures: 9-hexyl-9*H*-carbazole-3-carbaldehyde,^67^ 9-(2-ethylhexyl)-9*H*-carbazole, 9-(2-ethylhexyl)-9*H*-carbazole-3-carbaldehyde (**EHCzCHO**),^68^ 4-(3,6-*ditert*-butyl-9*H*-carbazol-9-yl)benzaldehyde (**PCzBCHO**),^69^ 4-(3,7-*ditert*-butyl-10*H*-phenothiazin-10-yl)benzaldehyde (**PPTBCHO**),^70^ 4-(3,6-dimethoxy-9*H*-carbazol-9-yl)benzaldehyde (**PCzMCHO**),^71^ 2,5-bis(9-hexylcarbazol-3-yl)thiazolo[5,4-*d*]thiazole (**TT-HCz**), and 4,4′-(thiazolo[5,4-*d*]thiazole-2,5-diyl)bis(N-(4-(hexyloxy)phenyl)-*N*-phenylaniline) (**TTP-DPA**).^72^

#### 2,5-Bis(9-(2-ethylhexyl)carbazol-3-yl)thiazolo[5,4-*d*]thiazole (TT-EHCz)

9-Hexylcarbazol-3-carbaldehyde 0.75 g (2.88 mmol) and dithiooxamide 0.175 g (1.44 mmol) were dissolved in 5 mL of DMF. The reaction was carried out at 160 °C for 8 h. After cooling to room temperature, the mixture was poured into distilled water. The yellow precipitate was filtered off and washed with water. The crude product was purified by column chromatography (*n*-hexane/ethyl acetate vol. ratio 10:1) and recrystallized from eluent to obtain yellow crystals of **TT-EHCz** (0.26 g, 26%), mp 152–154 °C.

^1^H NMR (400 MHz, CDCl_3_) δ 8.75 (s, 2H), 8.21 (d, *J* = 7.7 Hz, 2H), 8.11 (d, *J* = 8.6 Hz, 2H), 7.51 (t, *J* = 7.6 Hz, 2H), 7.44 (t, *J* = 9.2 Hz, 2H), 7.30 (t, *J* = 7.4 Hz, 2H), 4.20 (d, *J* = 7.3 Hz, 4H), 2.06–2.12 (m, 2H), 1.26–1.43 (m, 16H), 0.94 (t, *J* = 7.4 Hz, 6H), 0.87 (t, *J* = 7.1 Hz, 6H).

^13^C NMR (101 MHz, CDCl_3_) δ 167.76, 157.82, 142.28, 141.53, 141.49, 134.18, 126.36, 124.83, 125.31, 124.34, 123.32, 122.85, 120.78, 119.65, 118.65, 109.43, 55.51, 47.64, 39.43, 31.02, 28.81, 24.42, 23.05, 14.05, 10.93.

FTIR (cm^–1^): 3046, *v*(CH_ar_); 2953, 2921, 2850, *v*(CH_al_); 1627, 1596, 1446, 1412, *v*(C=C_ar_); 806, 745, 727, γ(C–H_ar_).

ESI*-*MS*(m*/*z*): calculated for C_44_H_48_N_4_S_2_ [M]^+^ = 696.33, found [M + H]^+^ = 697.20.

#### 2,5-Bis(4-(3,6-*ditert*-butyl-9*H*-carbazol-9-yl)phenyl)thiazolo[5,4-*d*]thiazole (TTP-CzB)

It was obtained from 4-(3,6-*ditert*-butyl-9*H*-carbazol-9-yl)benzaldehyde (0,62 g, 1,59 mmol) and dithiooxamide (0.095 g, 0.80 mmol) by the same procedure as **TT-EHCz**. Yield 0.21 g (32%), mp 276–278 °C.

^1^H NMR (400 MHz CDCl_3_) δ 8.20–8.25 (m, 2H); 8.15 (s, 4H); 8.02 (dd, *J*_*1*_ = 7.9 Hz, *J*_*2*_ = 5.4 Hz, 2H); 7.72 (t, *J* = 7.4 Hz, 2H); 7.44–7.51 (m, 8H); 7.19 (t, *J* = 8.2 Hz, 2H); 1,48 (s, 36H).

^13^C NMR (101 MHz CDCl_3_) δ 143.47; 140.46; 138.72; 132.07; 128.47; 128.38; 127.82; 126.82; 123.84; 123.77; 116.48; 116.41; 116.26; 109.27; 34.79; 32.01.

FTIR (cm^–1^): 3056, *v*(CH_ar_); 2953, 2900, 2863, *v*(CH_al_); 1601, 1487, 1470, 1448, *v*(C=C_ar_); 834, 812, 763, γ(C–H_ar_).

ESI*-*MS (*m*/*z*): calculated for C_56_H_56_N_4_S_2_ [M]^+^ = 848.39, found [M + H]^+^ = 849.55.

#### 2,5-Bis(4-(3,7-*ditert*-butyl-10*H*-phenothiazin-10-yl)phenyl)thiazolo[5,4-*d*]thiazole (TTP-PTB)

It was obtained from 4-(3,7-*ditert*-butyl-10*H*-phenothiazin-10-yl)benzaldehyde (0,56 g, 1,20 mmol) and dithiooxamide (0.11 g, 0.92 mmol) by the same procedure as **TT-EHCz**. Yield 0.20 g (24%).

^1^H NMR (400 MHz, CDCl_3_) δ 8.04 (d, *J* = 8.4 Hz, 4H); 7.33 (d, *J* = 8.4 Hz, 4H); 7.24 (d, *J* = 1.2 Hz, 4H); 7.09 (dd, *J*_*1*_ = 8.5 Hz, *J*_*2*_ = 1.2 Hz, 4H); 6.71 (d, *J* = 8.5 Hz, 4H); 1.28 (s, 36H).

^13^C NMR (101 MHz CDCl_3_) δ 168.34; 150.82, 147.31; 145.52; 140.43; 130.46, 128.12; 125.82; 124.76; 124.64; 124.03; 120.2; 34.33; 31.28.

FTIR (cm^–1^): 3052, *v*(CH_ar_); 2959, 2901, 2863, *v*(CH_al_); 1595, 1506, 1477, 1447, *v*(C=C_ar_); 1266, 1182, *v*(C–N_ar_); 864, 809, 724, 672 γ(C–H_ar_).

ESI**-**MS (*m*/*z*): calculated for C_56_H_56_N_4_S_4_ [M]^+^ = 912.34, found [M]^+^ = 912.90.

#### 2,5-Bis(4-(3,6-dimethoxy-9*H*-carbazol-9-yl)phenyl)thiazolo[5,4-*d*]thiazole (TTP-CzM)

It was obtained from 4-(3,6-dimethoxy-9*H*-carbazol-9-yl)benzaldehyde (0.70 g, 2.11 mmol) and dithiooxamide (0.12 g, 1.00 mmol) by the same procedure as **TT-EHCz**. Yield 0.25 g (33%), mp 215–217 °C.

^1^H NMR (400 MHz CDCl_3_) δ 8.23 (d, *J* = 8.1 Hz, 4H): 7.71 (d, *J* = 8.2 Hz, 4H); 7.57 (s, 4H); 7.44 (d, *J* = 8.9 Hz, 4H); 7.07 (dd, *J*_*1*_ = 9.0, *J*_*2*_ = 1.1 Hz, 4H); 3,97 (s, 12H).

^13^C NMR (101 MHz CDCl_3_) δ 168.11; 154.41; 140.24; 135.71; 131.96; 127.88; 126.73; 124.14; 115.32; 110.80; 103.07; 56.12.

FTIR (cm^–1^): 3025, *v*(CHar); 2932, 2892, 2836, *v*(CHal); 1600, 1521, 1468, 1429, *v*(C=Car); 833, 802, 785, γ(C-Har).

ESI**-**MS (*m*/*z*): calculated for C_44_H_32_N_4_S_2_ [M]^+^ = 744.19, found [M]^+^ = 744.49.

### Characterizations

A Bruker Avance III [400 MHz (^1^H), 101 MHz (^13^C)] apparatus was used at room temperature for recording of ^1^H NMR and ^13^C NMR spectra. Chemical shifts (δ) are reported in ppm referenced to tetramethylsilane or to the internal solvent signal. MS analysis was recorded on a Waters ZQ (Waters, Milford, Massachusetts). IR spectra were recorded in transmission mode using a Spectrum GX FTIR spectrometer (PerkinElmer) using potassium bromide for the formation of pellets. UV absorption spectra were measured with a Lambda 35 UV/vis spectrometer (PerkinElmer) and a Vertex 70 Bruker spectrometer equipped with an ATR attachment with a diamond crystal over frequencies of 600–3500 cm^–1^ with a resolution of 5 cm^–1^ over 32 scans. Fluorescence (FL) spectra were recorded using a PerkinElmer LS 55 DSC. Measurements were carried out using a DSC Q2000. TGA was performed on a TGA Q50. The TGA and DSC curves were recorded in a nitrogen atmosphere at a heating rate of 10 °C/min. The CV measurements were carried out by a three-electrode assembly cell from Bio-Logic SAS and a micro-Autolab Type III potentiostat–galvanostat. The working electrode was a glassy carbon with the surface of 0.12 cm^2^. The reference electrode and the counter electrode were Ag/Ag^+^ 0.01 M and Pt wire, respectively. The solutions with the concentration of 10^–3^ M of the compounds in argon-purged dichloromethane (Fluka) with tetrabulthylammonium perchlorate (TBAP; 0.1 M) as electrolyte were used for the CV measurements. Ionization potentials of films under an air atmosphere were measured by methods of photoelectron emission spectrometry. The samples for it were fabricated by the technique of thermal vacuum deposition onto fluorine-doped tin oxide-coated glass substrates. An ASBN-D130-CM-deep UV deuterium light source, a 6517B Keithley electrometer, and a CM110 1/8m monochromator were used for measurement of photoelectron emission spectra. Charge mobility values were studied by the time-of-flight (TOF) method. Various positive and negative external voltages (*U*) were applied to the samples using a 6517B electrometer (Keithley) to check hole and electron transports in the layers at the different electric fields. A TDS 3032C oscilloscope (Tektronix) was used to record the photocurrent transients of holes or electrons. Charge mobilities were estimated by the formula μ = *d*^2^/(*U* × *t*_tr_), where *t*_tr_ is the transit time, *d* is the thickness of a layer, and *U* is the applied voltage over the sample.

### Device Fabrication

The inverted PSCs were fabricated with the architecture of glass/ITO/HSL/Cs_0.18_FA_0.82_Pb(I_0.8_Br_0.2_)_3_/PEAI/PC_61_BM/BCP/Ag. Indium tin oxide (ITO) substrates were precleaned in an ultrasonic bath, sequentially with abstergent aqueous solution, deionized water, acetone, and isopropyl alcohol, for 20 min each, and then dried under a stream of N_2_. HSLs include 2PACz, compound **TTP-DPA**, and 2PACz/**TTP-DPA**. A novel HSL of thiazolo[5,4-*d*]thiazole was spin-cast (5000 rpm, 30 s) on ITO. A self-assembled layer of 2PACz as HSL was spin-cast (3000 rpm, 30 s) on ITO and then baked at 100 °C for 10 min. The 2PACz precursor solution was prepared by dissolving 2PACz in ethanol at a concentration of 1 mg/mL and was placed in an ultrasonic bath for 15 min before being used. For interfacial engineering between 2PACz and perovskite, the derivative of thiazolo[5,4-*d*]thiazole (i.e., **TTP-DPA**) was spin-cast (5000 rpm, 30 s) on the top of 2PACz. The thiazolo[5,4-*d*]thiazoles were prepared by dissolving in chloroform. The precursor^73^ solution for Cs_0.18_FA_0.82_Pb(I_0.8_Br_0.2_)_3_ was prepared by dissolving 172 mg of FAI, 30 mg of CsCl, 110 mg of PbBr_2_, and 354 mg of PbI_2_ in 1 mL of DMF:DMSO (4:1 in volume). The perovskite layer was deposited on the substrate in a two-step manner: first at 1000 rpm for 10 s and then at 5000 rpm for 30 s. During the second step, chlorobenzene (0.15 mL) was dropped on the spinning substrate; the sample was then annealed at 150 °C for 30 min. For the PEAI surface passivation, PEAI dissolved in IPA at a concentration of 1.5 mg/mL was spin-coated (5000 rpm, 30 s) on the top of the perovskite, followed by annealing at 100 °C for 1 min. For the electron transport layer, a solution of PC_61_BM in chlorobenzene (20 mg/mL) was spin-coated (2000 rpm, 30 s) on the top of the PEAI layer. For the hole-blocking layer, a solution of BCP in IPA (0.5 mg/mL) was spin-coated (6000 rpm, 10 s) onto the PC_61_BM layer. Finally, the devices were completed by evaporating Ag (100 nm) in a vacuum chamber; the active area of this electrode was fixed at 10 mm^2^, defined by the cross overlap of the 5 mm-wide-patterned ITO bar and the 2 mm-wide Ag bar deposited through a metal mask.

### Measurements

Device performance data were collected within a glovebox. The current–voltage (*I*–*V*) characteristics of the devices were measured employing a computer-controlled Keithley 2400 source measurement unit (SMU) and a Dyesol simulator (AAA Class Solar Simulators) under AM 1.5 illumination (100 mW/cm^2^). The light intensity was calibrated using a standard Si reference cell and a KG-5 filter. EQE spectra were recorded using an Enlitech QE-R spectral response measurement system to standardize the current densities of the devices. PL spectra were acquired using an Edinburgh FLS1000 PL spectrometer, employing an excitation wavelength of 550 nm. TCSPC spectra were also recorded using an Edinburgh FLS1000 PL spectrometer with a pulse laser featuring a wavelength of 447 nm. The laser operated with a 500 ns excitation duration. The comprehensive characterization platform Paios (Fluxim AG) was employed to assess the optoelectronic properties of PSCs, including photo-CELIV, TPC, and TPV measurements. Crystallinity information was obtained using a Malvern Panalytical Empyrean X-ray diffractometer with Cu Kα radiation (λ = 0.1542 nm) and a step size of 0.02°. The static contact angle measurements were recorded using a first 10 Å/FTA-1000B apparatus. SEM images were recorded on a HITACHI S-5200 SEM.

## Conclusions

Six donor-disubstituted TT derivatives were designed and synthesized. All the compounds exhibited high thermal stability. They showed 5% weight loss temperatures higher than 397 °C. TT containing two Cz or PTZ donor units with *tert*-butyl substituents showed the highest glass transition temperature values of 130 and 187 °C, respectively. The compounds were characterized by locally excited absorption bands caused by TT with a weak effect of intermolecular charge-transfer absorption in the visible region. Ionization potentials of the solid layers of the compounds estimated by photoelectron emission spectrometry in air ranged from 4.93 to 5.67 eV. Almost balanced hole and electron transports with mobilities of 3.97 × 10^–4^ and 6.69 × 10^–4^ cm^2^ V^–1^ s^–1^, respectively, were obtained for triphenylamino-substituted thiazolo[5,4-*d*]thiazole. The highest hole mobility reaching 1.63 × 10^–3^ cm^2^ V^–1^ s^–1^ was observed for the derivative of TT and dimethoxy-substituted phenyl Cz. The layer of TT with hexyloxy-substituted triphenylamino substituent (**TTP-DPA)** was used for the preparation of HSL and interlayer for wide-band-gap Cs_0.18_FA_0.82_Pb(I_0.8_Br_0.2_)_3_ PSCs. The optimized PSCs utilizing HSL of 2PAC/**TTP-DPA** HSL demonstrate maximum power conversion efficiencies of 19.1 and 37.0% under one-sun and 3000 K LED (1000 lx) illuminations, respectively, surpassing those observed control devices with HSL of 2PACz. These results underscore the potential of the D–A–D-type derivative of TT for interface modification as a practical and efficient approach to enhance passivation and contact properties of PSCs, highlighting their suitability as materials for HSLs.

## Supporting Information Available

DSC graphs of TT derivatives; TOF signals for vacuum-deposited films of TT compounds; additional photophysical data including PL spectra of TT materials in dilute toluene, THF, and chloroform solutions; ^1^H spectra of synthesized materials **TT-EHCz**, **TTP-CzB**, **TTP-PTB**, and **TTP-CzM**; water contact angle and DIM contact angle measurements; device performance of the PSCs, including PSCs and iPSCs (PDF)

## References

[ref1] Zheng X.; Li Z.; Zhang Y.; Chen M.; Liu T.; Xiao C.; Gao D.; Patel J. B.; Kuciauskas D.; Magomedov A.; Scheidt R. A.; Wang X.; Harvey S. P.; Dai Z.; Zhang C.; Morales D.; Pruett H.; Wieliczka B. M.; Kirmani A. R.; Padture N. P.; Graham K. R.; Yan Y.; Nazeeruddin M. K.; McGehee M. D.; Zhu Z.; Luther J. M. Co-Deposition of Hole-Selective Contact and Absorber for Improving the Processability of Perovskite Solar Cells. Nat. Energy 2023, 8 (5), 462–472. 10.1038/s41560-023-01227-6.

[ref2] Li K.; Zhang L.; Ma Y.; Gao Y.; Feng X.; Li Q.; Shang L.; Yuan N.; Ding J.; Jen A. K. Y.; You J.; Liu S. (. Au Nanocluster Assisted Microstructural Reconstruction for Buried Interface Healing for Enhanced Perovskite Solar Cell Performance. Adv Mater. 2023, 2310651 10.1002/adma.202310651. 38016668

[ref3] An Y.; Zhang N.; Zeng Z.; Cai Y.; Jiang W.; Qi F.; Ke L.; Lin F. R.; Tsang S.; Shi T.; Jen A. K. -Y.; Yip H. Optimizing Crystallization in Wide-Bandgap Mixed Halide Perovskites for High-Efficiency Solar Cells. Adv. Mater. 2024, 2306568 10.1002/adma.202306568. 37677058

[ref4] Yu S.; Xiong Z.; Zhou H.; Zhang Q.; Wang Z.; Ma F.; Qu Z.; Zhao Y.; Chu X.; Zhang X.; You J. Homogenized NiOx Nanoparticles for Improved Hole Transport in Inverted Perovskite Solar Cells. Science (1979) 2023, 382 (6677), 1399–1404. 10.1126/science.adj8858. 37995210

[ref5] Ma Q.; Wang Y.; Liu L.; Yang P.; He W.; Zhang X.; Zheng J.; Ma M.; Wan M.; Yang Y.; Zhang C.; Mahmoudi T.; Wu S.; Liu C.; Hahn Y.-B.; Mai Y. One-Step Dual-Additive Passivated Wide-Bandgap Perovskites to Realize 44.72%-Efficient Indoor Photovoltaics. Energy Environ. Sci. 2024, 17 (5), 1637–1644. 10.1039/D3EE04022D.

[ref6] Choi M. J.; Lee S. W.; Lee M.; Shin S. J.; Kim M.; Jeon G. G.; Yoon S. E.; Xiangyang F.; Lee B. R.; Seidel J.; Yun J. S.; Chang D. W.; Kim J. H. Strategic Approach for Achieving High Indoor Efficiency of Perovskite Solar Cells: Frustration of Charge Recombination by Dipole Induced Homogeneous Charge Distribution. Chemical Engineering Journal 2023, 454, 140284 10.1016/j.cej.2022.140284.

[ref7] Li Y.; Li R.; Lin Q. Engineering the Non-Radiative Recombination of Mixed-Halide Perovskites with Optimal Bandgap for Indoor Photovoltaics. Small 2022, 18 (26), 2202028 10.1002/smll.202202028. 35616062

[ref8] Wang K.; Lu H.; Li M.; Chen C.; Bo Zhang D.; Chen J.; Wu J.; Zhou Y.; Wang X.; Su Z.; Shi Y.; Tian Q.; Ni Y.; Gao X.; Zakeeruddin S. M.; Grätzel M.; Wang Z.; Liao L. Ion–Dipole Interaction Enabling Highly Efficient CsPbI3 Perovskite Indoor Photovoltaics. Adv. Mater. 2023, 35 (31), 2210106 10.1002/adma.202210106. 37286198

[ref9] Wang H.; Zhang W.; Wang B.; Yan Z.; Chen C.; Hua Y.; Wu T.; Wang L.; Xu H.; Cheng M. Modulating Buried Interface with Multi-Fluorine Containing Organic Molecule toward Efficient NiO -Based Inverted Perovskite Solar Cell. Nano Energy 2023, 111, 108363 10.1016/j.nanoen.2023.108363.

[ref10] Li C.; Zhang Y.; Zhang X.; Zhang P.; Yang X.; Chen H. Efficient Inverted Perovskite Solar Cells with a Fill Factor Over 86% via Surface Modification of the Nickel Oxide Hole Contact. Adv. Funct Mater. 2023, 33 (13), 2214774 10.1002/adfm.202214774.

[ref11] Jiang B.; Gao Z.; Lung C.; Shi Z.; Du H.; Su Y.; Shih H.; Lee K.; Hung H.; Chan C. K.; Chen C.; Wong K. Enhancing the Efficiency of Indoor Perovskite Solar Cells through Surface Defect Passivation with Coplanar Heteroacene Cored A–D–A-type Molecules. Adv. Funct Mater. 2024, 2312819 10.1002/adfm.202312819.

[ref12] Guo R.; Wang X.; Jia X.; Guo X.; Li J.; Li Z.; Sun K.; Jiang X.; Alvianto E.; Shi Z.; Schwartzkopf M.; Müller-Buschbaum P.; Hou Y. Refining the Substrate Surface Morphology for Achieving Efficient Inverted Perovskite Solar Cells. Adv. Energy Mater. 2023, 13 (43), 2302280 10.1002/aenm.202302280.

[ref13] Kim S. Y.; Cho S. J.; Byeon S. E.; He X.; Yoon H. J. Self-Assembled Monolayers as Interface Engineering Nanomaterials in Perovskite Solar Cells. Adv. Energy Mater. 2020, 10 (44), 2002606 10.1002/aenm.202002606.

[ref14] Jiang W.; Liu M.; Li Y.; Lin F. R.; Jen A. K.-Y. Rational Molecular Design of Multifunctional Self-Assembled Monolayers for Efficient Hole Selection and Buried Interface Passivation in Inverted Perovskite Solar Cells. Chem. Sci. 2024, 15 (8), 2778–2785. 10.1039/D3SC05485C. 38404377 PMC10882494

[ref15] Ullah A.; Park K. H.; Nguyen H. D.; Siddique Y.; Shah S. F. A.; Tran H.; Park S.; Lee S. I.; Lee K.; Han C.; Kim K.; Ahn S.; Jeong I.; Park Y. S.; Hong S. Novel Phenothiazine-Based Self-Assembled Monolayer as a Hole Selective Contact for Highly Efficient and Stable P-i-n Perovskite Solar Cells. Adv. Energy Mater. 2022, 12 (2), 2103175 10.1002/aenm.202103175.

[ref16] Castriotta L. A.; Infantino R.; Vesce L.; Stefanelli M.; Dessì A.; Coppola C.; Calamante M.; Reginato G.; Mordini A.; Sinicropi A.; Di Carlo A.; Zani L. Stable Methylammonium-Free P-i-n Perovskite Solar Cells and Mini-Modules with Phenothiazine Dimers as Hole-Transporting Materials. Energy Environ. Mater. 2023, 6 (6), e12455 10.1002/eem2.12455.

[ref17] Wang G.; Zheng J.; Duan W.; Yang J.; Mahmud M. A.; Lian Q.; Tang S.; Liao C.; Bing J.; Yi J.; Leung T. L.; Cui X.; Chen H.; Jiang F.; Huang Y.; Lambertz A.; Jankovec M.; Topič M.; Bremner S.; Zhang Y.-Z.; Cheng C.; Ding K.; Ho-Baillie A. Molecular Engineering of Hole-Selective Layer for High Band Gap Perovskites for Highly Efficient and Stable Perovskite-Silicon Tandem Solar Cells. Joule 2023, 7 (11), 2583–2594. 10.1016/j.joule.2023.09.007.

[ref18] Sholihah N.; Cheng H.-C.; Wang J.-C.; Ni J.-S.; Yu Y.-Y.; Chen C.-P.; Chen Y.-C. Passivation of Inverted Perovskite Solar Cells by Trifluoromethyl-Group-Modified triphenylamine Dibenzofulvene Hole Transporting Interfacial Layers. J. Phys. Chem. C 2023, 127 (13), 6167–6178. 10.1021/acs.jpcc.3c00113.

[ref19] Truong M. A.; Funasaki T.; Ueberricke L.; Nojo W.; Murdey R.; Yamada T.; Hu S.; Akatsuka A.; Sekiguchi N.; Hira S.; Xie L.; Nakamura T.; Shioya N.; Kan D.; Tsuji Y.; Iikubo S.; Yoshida H.; Shimakawa Y.; Hasegawa T.; Kanemitsu Y.; Suzuki T.; Wakamiya A. Tripodal Triazatruxene Derivative as a Face-On Oriented Hole-Collecting Monolayer for Efficient and Stable Inverted Perovskite Solar Cells. J. Am. Chem. Soc. 2023, 145 (13), 7528–7539. 10.1021/jacs.3c00805. 36947735

[ref20] Cao Q.; Wang T.; Pu X.; He X.; Xiao M.; Chen H.; Zhuang L.; Wei Q.; Loi H.; Guo P.; Kang B.; Feng G.; Zhuang J.; Feng G.; Li X.; Yan F. Co-Self-Assembled Monolayers Modified NiOx for Stable Inverted Perovskite Solar Cells. Adv. Mater. 2024, 2311970 10.1002/adma.202311970. 38198824

[ref21] Liu M.; Bi L.; Jiang W.; Zeng Z.; Tsang S.; Lin F. R.; Jen A. K. -Y. Compact Hole-Selective Self-Assembled Monolayers Enabled by Disassembling Micelles in Solution for Efficient Perovskite Solar Cells. Adv. Mater. 2023, 35 (46), 2304415 10.1002/adma.202304415. 37487572

[ref22] Bevk D.; Marin L.; Lutsen L.; Vanderzande D.; Maes W. Thiazolo[5,4-d]Thiazoles-Promising Building Blocks in the Synthesis of Semiconductors for Plastic Electronics. RSC Adv. 2013, 7, 11418–11431. 10.1039/c3ra40851e.

[ref23] Lim D. H.; Jang S. Y.; Kang M.; Lee S.; Kim Y. A.; Heo Y. J.; Lee M. H.; Kim D. Y. A Systematic Study on Molecular Planarity and D-A Conformation in Thiazolothiazole- and Thienylenevinylene-Based Copolymers for Organic Field-Effect Transistors. J. Mater. Chem. C Mater. 2017, 5 (39), 10126–10132. 10.1039/C7TC02273E.

[ref24] Dessì A.; Calamante M.; Sinicropi A.; Parisi M. L.; Vesce L.; Mariani P.; Taheri B.; Ciocca M.; Di Carlo A.; Zani L.; Mordini A.; Reginato G. Thiazolo[5,4-d]thiazole-Based Organic Sensitizers with Improved Spectral Properties for Application in Greenhouse-Integrated Dye-Sensitized Solar Cells. Sustain Energy Fuels 2020, 4 (5), 2309–2321. 10.1039/D0SE00124D.

[ref25] Sathiyan G.; Ranjan R.; Ranjan S.; Garg A.; Gupta R. K.; Singh A. Dicyanovinylene and Thiazolo[5,4-d]thiazole Core Containing D-A-D Type Hole-Transporting Materials for Spiro-OMeTAD-Free Perovskite Solar Cell Applications with Superior Atmospheric Stability. ACS Appl. Energy Mater. 2019, 2 (10), 7609–7618. 10.1021/acsaem.9b01598.

[ref26] Sayresmith N. A.; Saminathan A.; Sailer J. K.; Patberg S. M.; Sandor K.; Krishnan Y.; Walter M. G. Photostable Voltage-Sensitive Dyes Based on Simple, Solvatofluorochromic, Asymmetric Thiazolothiazoles. J. Am. Chem. Soc. 2019, 141 (47), 18780–18790. 10.1021/jacs.9b08959. 31660737

[ref27] Farokhi A.; Shahroosvand H.; Monache G. D.; Pilkington M.; Nazeeruddin M. K. The Evolution of triphenylamine Hole Transport Materials for Efficient Perovskite Solar Cells. Chem. Soc. Rev. 2022, 30, 5974–6064. 10.1039/d1cs01157j. 35770784

[ref28] Zhang D.; Yang T.; Xu H.; Miao Y.; Chen R.; Shinar R.; Shinar J.; Wang H.; Xu B.; Yu J. triphenylamine/Benzothiadiazole-Based Compounds for Non-Doped Orange and Red Fluorescent OLEDs with High Efficiencies and Low Efficiency Roll-Off. J. Mater. Chem. C Mater. 2021, 9 (14), 4921–4926. 10.1039/D1TC00249J.

[ref29] Gangadhar P. S.; Reddy G.; Prasanthkumar S.; Giribabu L. Phenothiazine Functional Materials for Organic Optoelectronic Applications. Phys. Chem. Chem. Phys. 2021, 14969–14996. 10.1039/d1cp01185e. 34231592

[ref30] Gao L.; Schloemer T. H.; Zhang F.; Chen X.; Xiao C.; Zhu K.; Sellinger A. Carbazole-Based Hole-Transport Materials for High-Efficiency and Stable Perovskite Solar Cells. ACS Appl. Energy Mater. 2020, 3 (5), 4492–4498. 10.1021/acsaem.0c00179.

[ref31] Ledwon P. Recent Advances of Donor-Acceptor Type Carbazole-Based Molecules for Light Emitting Applications. Org. Electron. 2019, 105422 10.1016/j.orgel.2019.105422.

[ref32] Magomedov A.; Paek S.; Gratia P.; Kasparavicius E.; Daskeviciene M.; Kamarauskas E.; Gruodis A.; Jankauskas V.; Kantminiene K.; Cho K. T.; Rakstys K.; Malinauskas T.; Getautis V.; Nazeeruddin M. K. Diphenylamine-Substituted Carbazole-Based Hole Transporting Materials for Perovskite Solar Cells: Influence of Isomeric Derivatives. Adv. Funct Mater. 2018, 28 (9), 1704351 10.1002/adfm.201704351.

[ref33] Thokala S.; Singh S. P. Phenothiazine-Based Hole Transport Materials for Perovskite Solar Cells. ACS Omega 2020, 5 (11), 5608–5619. 10.1021/acsomega.0c00065. 32226836 PMC7097910

[ref34] Rybakiewicz R.; Zagorska M.; Pron A. triphenylamine-Based Electroactive Compounds: Synthesis, Properties and Application to Organic Electronics. Chemical Papers 2017, 71 (2), 243–268. 10.1007/s11696-016-0097-0.

[ref35] Keruckas J.; Lygaitis R.; Simokaitiene J.; Grazulevicius J. V.; Jankauskas V.; Sini G. Influence of Methoxy Groups on the Properties of 1,1-Bis(4-Aminophenyl)Cyclohexane Based Arylamines: Experimental and Theoretical Approach. J. Mater. Chem. 2012, 22 (7), 3015 10.1039/c2jm14387a.

[ref36] He Y.; Zhang C.; Yan H.; Chai Y.; Zhou D. A Simple Strategy for Obtaining Aggregation-Induced Delayed Fluorescence Material Achieving Nearly 20% External Quantum Efficiency for Non-Doped Solution-Processed OLEDs. Chemical Engineering Journal 2023, 476, 146675 10.1016/j.cej.2023.146675.

[ref37] Zhang J.; Yang J.; Dai R.; Sheng W.; Su Y.; Zhong Y.; Li X.; Tan L.; Chen Y. Elimination of Interfacial Lattice Mismatch and Detrimental Reaction by Self-Assembled Layer Dual-Passivation for Efficient and Stable Inverted Perovskite Solar Cells. Adv. Energy Mater. 2022, 12 (18), 2103674 10.1002/aenm.202103674.

[ref38] Farag A.; Feeney T.; Hossain I. M.; Schackmar F.; Fassl P.; Küster K.; Bäuerle R.; Ruiz-Preciado M. A.; Hentschel M.; Ritzer D. B.; Diercks A.; Li Y.; Nejand B. A.; Laufer F.; Singh R.; Starke U.; Paetzold U. W. Evaporated Self-Assembled Monolayer Hole Transport Layers: Lossless Interfaces in P-i-n Perovskite Solar Cells. Adv. Energy Mater. 2023, 13 (8), 2203982 10.1002/aenm.202203982.

[ref39] Zhang X.; Qiu W.; Apergi S.; Singh S.; Marchezi P.; Song W.; Sternemann C.; Elkhouly K.; Zhang D.; Aguirre A.; Merckx T.; Krishna A.; Shi Y.; Bracesco A.; van Helvoirt C.; Bens F.; Zardetto V.; D’Haen J.; Yu A.; Brocks G.; Aernouts T.; Moons E.; Tao S.; Zhan Y.; Kuang Y.; Poortmans J. Minimizing the Interface-Driven Losses in Inverted Perovskite Solar Cells and Modules. ACS Energy Lett. 2023, 8 (6), 2532–2542. 10.1021/acsenergylett.3c00697.

[ref40] Zhang S.; Ye F.; Wang X.; Chen R.; Zhang H.; Zhan L.; Jiang X.; Li Y.; Ji X.; Liu S.; Yu M.; Yu F.; Zhang Y.; Wu R.; Liu Z.; Ning Z.; Neher D.; Han L.; Lin Y.; Tian H.; Chen W.; Stolterfoht M.; Zhang L.; Zhu W.-H.; Wu Y. Minimizing Buried Interfacial Defects for Efficient Inverted Perovskite Solar Cells. Science (1979) 2023, 380 (6643), 404–409. 10.1126/science.adg3755. 37104579

[ref41] Chiu Y.-L.; Li C.-W.; Kang Y.-H.; Lin C.-W.; Lu C.-W.; Chen C.-P.; Chang Y. J. Dual-Functional Enantiomeric Compounds as Hole-Transporting Materials and Interfacial Layers in Perovskite Solar Cells. ACS Appl. Mater. Interfaces 2022, 14 (22), 26135–26147. 10.1021/acsami.2c03025. 35634977

[ref42] Maddala S.; Chung C.-L.; Wang S.-Y.; Kollimalayan K.; Hsu H.-L.; Venkatakrishnan P.; Chen C.-P.; Chang Y. J. Forming a Metal-Free Oxidatively Coupled Agent, Bicarbazole, as a Defect Passivation for HTM and an Interfacial Layer in a p–i–n Perovskite Solar Cell Exhibits Nearly 20% Efficiency. Chem. Mater. 2020, 32 (1), 127–138. 10.1021/acs.chemmater.9b02720.

[ref43] Zhang Z.; Qiao L.; Meng K.; Long R.; Chen G.; Gao P. Rationalization of Passivation Strategies toward High-Performance Perovskite Solar Cells. Chem. Soc. Rev. 2023, 52 (1), 163–195. 10.1039/D2CS00217E. 36454225

[ref44] Ou Y.; Huang H.; Shi H.; Li Z.; Chen Z.; Mateen M.; Lu Z.; Chi D.; Huang S. Collaborative Interfacial Modification and Surficial Passivation for High-Efficiency MA-Free Wide-Bandgap Perovskite Solar Cells. Chemical Engineering Journal 2023, 469, 143860 10.1016/j.cej.2023.143860.

[ref45] Wang K.; Huang S.; Zhang Y.; Zhao S.; Zhang H.; Wang Y. Multicolor Fluorescence and Electroluminescence of an ICT-Type Organic Solid Tuned by Modulating the Accepting Nature of the Central Core. Chem. Sci. 2013, 4 (8), 3288 10.1039/c3sc51091c.

[ref46] Khan F.; Volyniuk L.; Ghasemi M.; Volyniuk D.; Grazulevicius J. V.; Misra R. Efficient Monomolecular White Emission of Phenothiazine Boronic Ester Derivatives with Room Temperature Phosphorescence. J. Mater. Chem. C Mater. 2022, 10 (28), 10347–10355. 10.1039/D2TC01612E.

[ref47] McMahon D. P.; Troisi A. Evaluation of the External Reorganization Energy of Polyacenes. J. Phys. Chem. Lett. 2010, 1 (6), 941–946. 10.1021/jz1001049.

[ref48] Senevirathna W.; Daddario C. M.; Sauvé G. Density Functional Theory Study Predicts Low Reorganization Energies for Azadipyrromethene-Based Metal Complexes. J. Phys. Chem. Lett. 2014, 5 (5), 935–941. 10.1021/jz402735c. 26274092

[ref49] Yu H. S.; He X.; Li S. L.; Truhlar D. G. MN15: A Kohn–Sham Global-Hybrid Exchange–Correlation Density Functional with Broad Accuracy for Multi-Reference and Single-Reference Systems and Noncovalent Interactions. Chem. Sci. 2016, 7 (8), 5032–5051. 10.1039/C6SC00705H. 30155154 PMC6018516

[ref50] Marenich A. V.; Cramer C. J.; Truhlar D. G. Universal Solvation Model Based on Solute Electron Density and on a Continuum Model of the Solvent Defined by the Bulk Dielectric Constant and Atomic Surface Tensions. J. Phys. Chem. B 2009, 113 (18), 6378–6396. 10.1021/jp810292n. 19366259

[ref51] Cammi R.; Mennucci B. Linear Response Theory for the Polarizable Continuum Model. J. Chem. Phys. 1999, 110 (20), 9877–9886. 10.1063/1.478861.

[ref52] Frisch M.J.; Trucks G.W.; Schlegel H.B.; Scuseria G.E.; Rob M.A.; Cheeseman J.R.; Scalmani G.; Barone V.; Petersson G.A.; Nakatsuji H.; Li X.; Caricato M.; Marenich A.V.; Bloino J.; Janesko B. G.; Gomperts R.; Mennucci B.; Hratchian H. P.; Ortiz J.V.; Izmaylov A. F.; Sonnenberg J. L.; Williams-Young D.; Ding F.; Lipparini F.; Egidi F.; Goings J.; Peng B.; Petrone A.; Henderson T.; Ranasinghe D.; Zakrzewski V. G.; Gao J.; Rega N.; Zheng G.; Liang W.; Hada M.; Ehara M.; Toyota K.; Fukuda R.; Hasegawa J.; Ishida M.; Nakajima T.; Honda Y.; Kitao O.; Nakai H.; Vreven T.; Throssell K.; Montgomery J. A.; Peralta J. E.; Ogliaro F.; Bearpark M. J.; Heyd J.J.; Brothers E. N.; Kudin K. N.; Staroverov V. N.; Keith T. A.; Kobayashi R.; Normand J.; Raghavachari K.; Rendell A. P.; Burant J. C.; Iyengar S. S.; Tomasi J.; Cossi M.; Millam J.M.; Klene M.; Adamo C.; Cammi R.; Ochterski J. W.; Martin R. L.; Morokuma K.; Farkas O.; Foresman J. B.; Fox D. J. Gaussian, Inc., Wallingford CT, “Gaussian 16, Revision B, Gaussian, Inc.*:* Wallingford, CT, 2016.

[ref53] Lu T.; Chen F. Multiwfn: A Multifunctional Wavefunction Analyzer. J. Comput. Chem. 2012, 33 (5), 580–592. 10.1002/jcc.22885. 22162017

[ref54] Cui H.; Huang L.; Zhou S.; Wang C.; Hu X.; Guan H.; Wang S.; Shao W.; Pu D.; Dong K.; Zhou J.; Jia P.; Wang W.; Tao C.; Ke W.; Fang G. Lead Halide Coordination Competition at Buried Interfaces for Low VOC-Deficits in Wide-Bandgap Perovskite Solar Cells. Energy Environ. Sci. 2023, 16 (12), 5992–6002. 10.1039/D3EE02818F.

[ref55] Liu S.-C.; Lin H.-Y.; Hsu S.-E.; Wu D.-T.; Sathasivam S.; Daboczi M.; Hsieh H.-J.; Zeng C.-S.; Hsu T.-G.; Eslava S.; Macdonald T. J.; Lin C.-T. Highly Reproducible Self-Assembled Monolayer Based Perovskite Solar Cells via Amphiphilic Polyelectrolyte. J. Mater. Chem. A Mater. 2024, 12 (5), 2856–2866. 10.1039/D3TA04512A.

[ref56] Jiang B.-H.; Peng Y.-J.; Chen C.-P. Simple Structured Polyetheramines, Jeffamines, as Efficient Cathode Interfacial Layers for Organic Photovoltaics Providing Power Conversion Efficiencies up to 9.1%. J. Mater. Chem. A Mater. 2017, 5 (21), 10424–10429. 10.1039/C7TA02954C.

[ref57] Pan T.; Zhou W.; Wei Q.; Peng Z.; Wang H.; Jiang X.; Zang Z.; Li H.; Yu D.; Zhou Q.; Pan M.; Zhou W.; Ning Z. Surface-Energy-Regulated Growth of Α-Phase Cs 0.03 FA 0.97 PbI3 for Highly Efficient and Stable Inverted Perovskite Solar Cells. Adv. Mater. 2023, 2208522 10.1002/adma.202208522. 36692303

[ref58] Zhang Z.; Wang J.; Liang J.; Zheng Y.; Wu X.; Tian C.; Sun A.; Huang Y.; Zhou Z.; Yang Y.; Liu Y.; Tang C.; Chen Z.; Chen C. Organizing Uniform Phase Distribution in Methylammonium-Free 1.77 EV Wide-Bandgap Inverted Perovskite Solar Cells. Small 2023, 19 (40), 2303213 10.1002/smll.202303213. 37269195

[ref59] Shen X.; Gallant B. M.; Holzhey P.; Smith J. A.; Elmestekawy K. A.; Yuan Z.; Rathnayake P. V. G. M.; Bernardi S.; Dasgupta A.; Kasparavicius E.; Malinauskas T.; Caprioglio P.; Shargaieva O.; Lin Y.; McCarthy M. M.; Unger E.; Getautis V.; Widmer-Cooper A.; Herz L. M.; Snaith H. J. Chloride-Based Additive Engineering for Efficient and Stable Wide-Bandgap Perovskite Solar Cells. Adv. Mater. 2023, 35 (30), 2211742 10.1002/adma.202211742. 37191054

[ref60] Hung C.-M.; Wu C.-C.; Tsao P.-H.; Lung C.-D.; Wang C.-H.; Ni I.-C.; Chu C.-C.; Cheng C.-H.; Kuang W.-Y.; Wu C.-I.; Chen H.-C.; Chan Y.-T.; Chou P.-T. Functionalization of Donor−π–Acceptor Hole Transport Materials Enhances Crystallization and Defect Passivation in Inverted Perovskite Solar Cells: Achieving Power Conversion Efficiency > 21% (Area: 1.96 Cm2 and Impressive Stability. Adv. Energy Sustain. Res. 2023, 4 (10), 2300042 10.1002/aesr.202300042.

[ref61] Li Z.; Sun X.; Zheng X.; Li B.; Gao D.; Zhang S.; Wu X.; Li S.; Gong J.; Luther J. M.; Li Z.; Zhu Z. Stabilized Hole-Selective Layer for High-Performance Inverted p-i-n Perovskite Solar Cells. Science (1979) 2023, 382 (6668), 284–289. 10.1126/science.ade9637. 37856581

[ref62] Zhu J.; Luo Y.; He R.; Chen C.; Wang Y.; Luo J.; Yi Z.; Thiesbrummel J.; Wang C.; Lang F.; Lai H.; Xu Y.; Wang J.; Zhang Z.; Liang W.; Cui G.; Ren S.; Hao X.; Huang H.; Wang Y.; Yao F.; Lin Q.; Wu L.; Zhang J.; Stolterfoht M.; Fu F.; Zhao D. A Donor–Acceptor-Type Hole-Selective Contact Reducing Non-Radiative Recombination Losses in Both Subcells towards Efficient All-Perovskite Tandems. Nat. Energy 2023, 8 (7), 714–724. 10.1038/s41560-023-01274-z.

[ref63] Wang R.; Xue J.; Meng L.; Lee J.-W.; Zhao Z.; Sun P.; Cai L.; Huang T.; Wang Z.; Wang Z.-K.; Duan Y.; Yang J. L.; Tan S.; Yuan Y.; Huang Y.; Yang Y. Caffeine Improves the Performance and Thermal Stability of Perovskite Solar Cells. Joule 2019, 3 (6), 1464–1477. 10.1016/j.joule.2019.04.005.

[ref64] Noh Y. W.; Ha J. M.; Son J. G.; Han J.; Lee H.; Kim D. W.; Jee M. H.; Shin W. G.; Cho S.; Kim J. Y.; Song M. H.; Woo H. Y. Improved Photovoltaic Performance and Stability of Perovskite Solar Cells by Adoption of an N-Type Zwitterionic Cathode Interlayer. Mater. Horiz 2024, 10.1039/D4MH00253A. 38567487

[ref65] Ahmed S. F.; Islam N.; Kumar P. S.; Hoang A. T.; Mofijur M.; Inayat A.; Shafiullah G. M.; Vo D.-V. N.; Badruddin I. A.; Kamangar S. Perovskite Solar Cells: Thermal and Chemical Stability Improvement, and Economic Analysis. Mater. Today Chem. 2023, 27, 101284 10.1016/j.mtchem.2022.101284.

[ref66] Harwood L. M.; Moody C. J.. Experimental Organic Chemistry: Principles and Practice; Blackwell: Oxford, 1989.

[ref67] Wu C.-S.; Fang S.-W.; Chen Y. Solution-Processable Hole-Transporting Material Containing Fluorenyl Core and Triple-Carbazolyl Terminals: Synthesis and Application to Enhancement of Electroluminescence. Phys. Chem. Chem. Phys. 2013, 15 (36), 15121 10.1039/c3cp52087k. 23925249

[ref68] Yang Y.; Xue M.; Marshall L. J.; de Mendoza J. Hydrogen-Bonded Cyclic Tetramers Based on Ureidopyrimidinones Attached to a 3,6-Carbazolyl Spacer. Org. Lett. 2011, 13 (12), 3186–3189. 10.1021/ol200946b. 21608985

[ref69] Fu B.; Dong X.; Yu X.; Zhang Z.; Sun L.; Zhu W.; Liang X.; Xu H. Meso-Borneol- Andmeso-Carbazole-Substituted Porphyrins: Multifunctional Chromophores with Tunable Electronic Structures and Antitumor Activities. New J. Chem. 2021, 45 (4), 2141–2146. 10.1039/D0NJ02954H.

[ref70] Ono K.; Yamaguchi T.; Tomura M. Structure and Photovoltaic Properties of (*E*)-2-Cyano-3-[4-(diphenylamino)Phenyl]Acrylic Acid Substituted by *Tert* -Butyl Groups. Chem. Lett. 2010, 39 (8), 864–866. 10.1246/cl.2010.864.

[ref71] Jia X.; Yu H.; Chen J.; Gao W.; Fang J.; Qin Y.; Hu X.; Shao G. Stimuli-Responsive Properties of Aggregation-Induced-Emission Compounds Containing a 9,10-Distyrylanthracene Moiety. Chem. - Eur. J. 2018, 24 (71), 19053–19059. 10.1002/chem.201804315. 30222213

[ref72] Dabuliene A.; Dainyte A.; Andruleviciene V.; Lygaitis R.; Punniyakoti S. M.; Tomkeviciene A.; Velasco D.; Obushak M.; Grazulevicius J. V. Low-Molar-Mass and Oligomeric Derivatives of Carbazole and triphenylamine Containing Thiazolo[5,4-d]thiazole Moieties. Polym. Bull. 2023. 80 1477 10.1007/s00289-022-04118-0.

[ref73] Prochowicz D.; Runjhun R.; Tavakoli M. M.; Yadav P.; Saski M.; Alanazi A. Q.; Kubicki D. J.; Kaszkur Z.; Zakeeruddin S. M.; Lewiński J.; Grätzel M. Engineering of Perovskite Materials Based on Formamidinium and Cesium Hybridization for High-Efficiency Solar Cells. Chem. Mater. 2019, 31 (5), 1620–1627. 10.1021/acs.chemmater.8b04871.

