# Supplementary material for: Enhancement of Efficiency of Perovskite Solar Cells with Hole-Selective Layers of Rationally Designed Thiazolo[5,4-d]thiazole Derivatives
Source: ACS Appl Mater Interfaces. 2024 May 29;16(23):30239–54. doi: 10.1021/acsami.4c04105 (PMC11181279; doi:10.1021/acsami.4c04105)
Supplement: Supplementary file 1 — am4c04105_si_001.pdf [file am4c04105_si_001.pdf]

Supporting Information

Enhancement of Efficiency of Perovskite Solar Cells with Hole Selective Layers of Rationally Designed Thiazolo[5,4-d]thiazole Derivatives

Asta Dabuliene[1‡], Zhong-En Shi [2‡], Karolis Leitonas[1], Chien-Yu, Lung[2], Dmytro Volyniuk, Khushdeep Kaur[1], Vitaly Matulis[3], Dmitry Lyakhov[4], Dominik Michels[4], Chih-Ping Chen[2,5*], Juozas Vidas Grazulevicius[1*]

[1]Department of Polymer Chemistry and Technology, Kaunas University of Technology, Baršausko Str. 59, LT-51423, Kaunas, Lithuania; e-mail: juozas.grazulevicius@ktu.lt

[2]Department of Materials Engineering and Organic Electronics Research Center, Ming Chi University of Technology, New Taipei City 243, Taiwan

[3]Belarusian State University, 220030 Minsk, Republic of Belarus

[4]Computer, Electrical and Mathematical Science and Engineering Division, 4700 King Abdullah University of Science and Technology, Thuwal 23955-6900, Saudi Arabia

[5]College of Engineering and Center for Sustainability and Energy Technologies, Chang Gung University, Taoyuan City 33302, Taiwan

[#] A. Dabuliene and Z.E. Shi. contributed equally to this work.

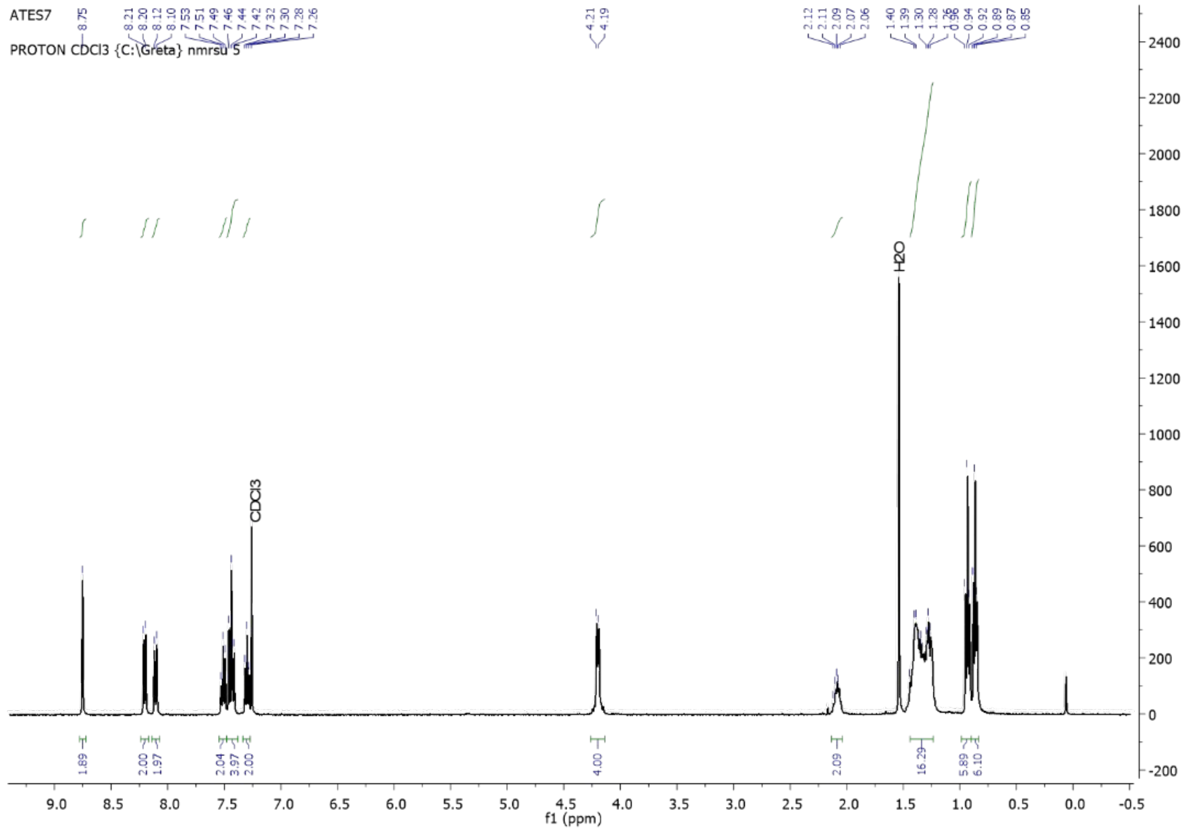

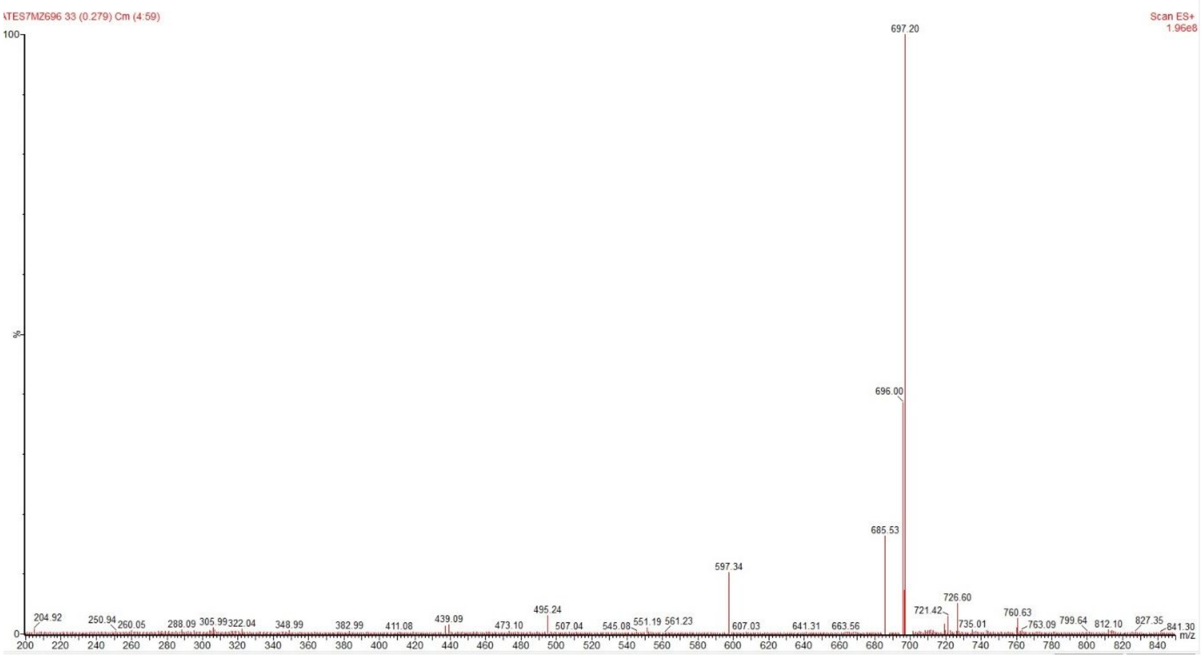

Fig. S1. ^{1}H-NMR and MS spectra of **TT-EHCz**

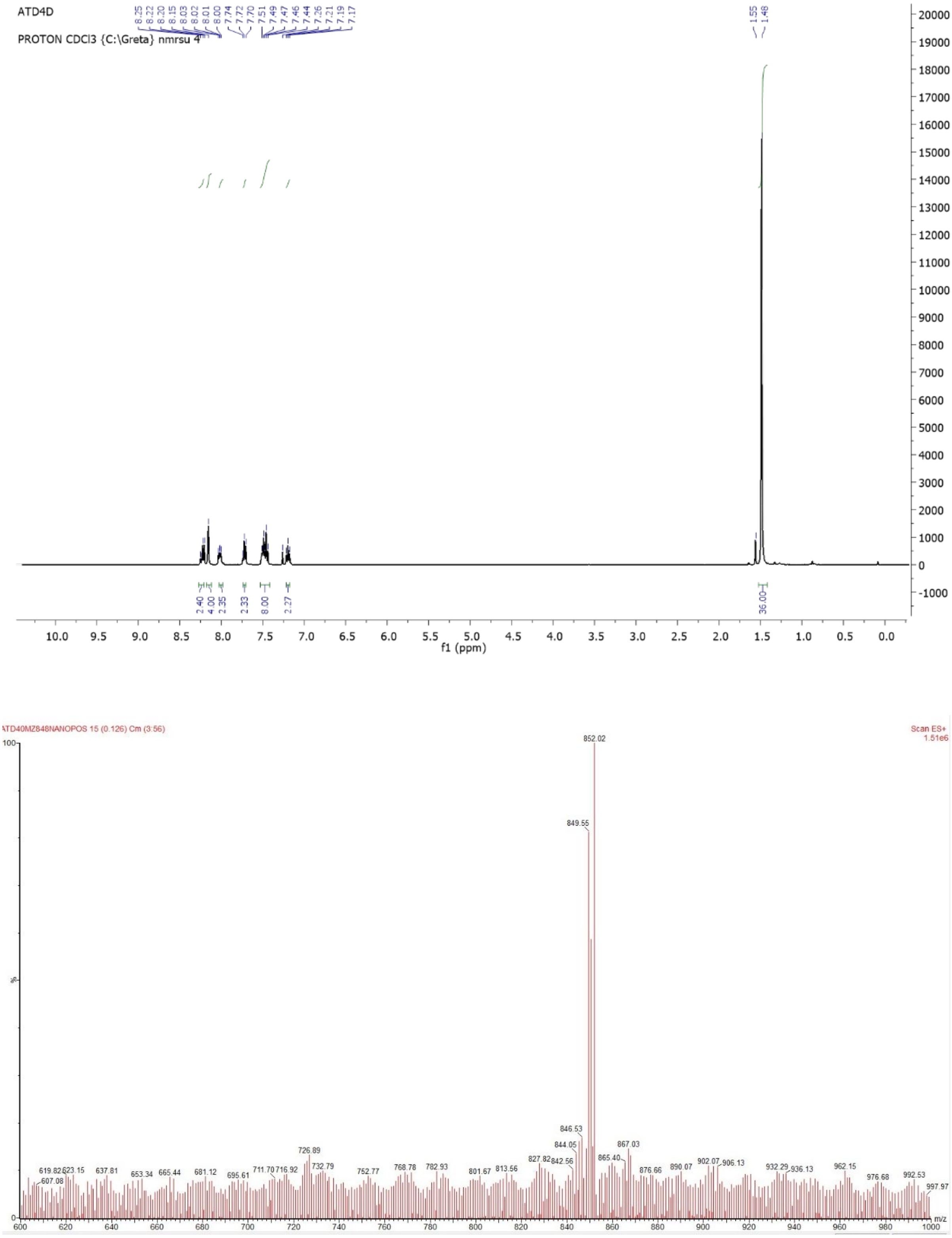

Fig. S2. ^{1}H-NMR and MS spectra of **TTP-CzB**

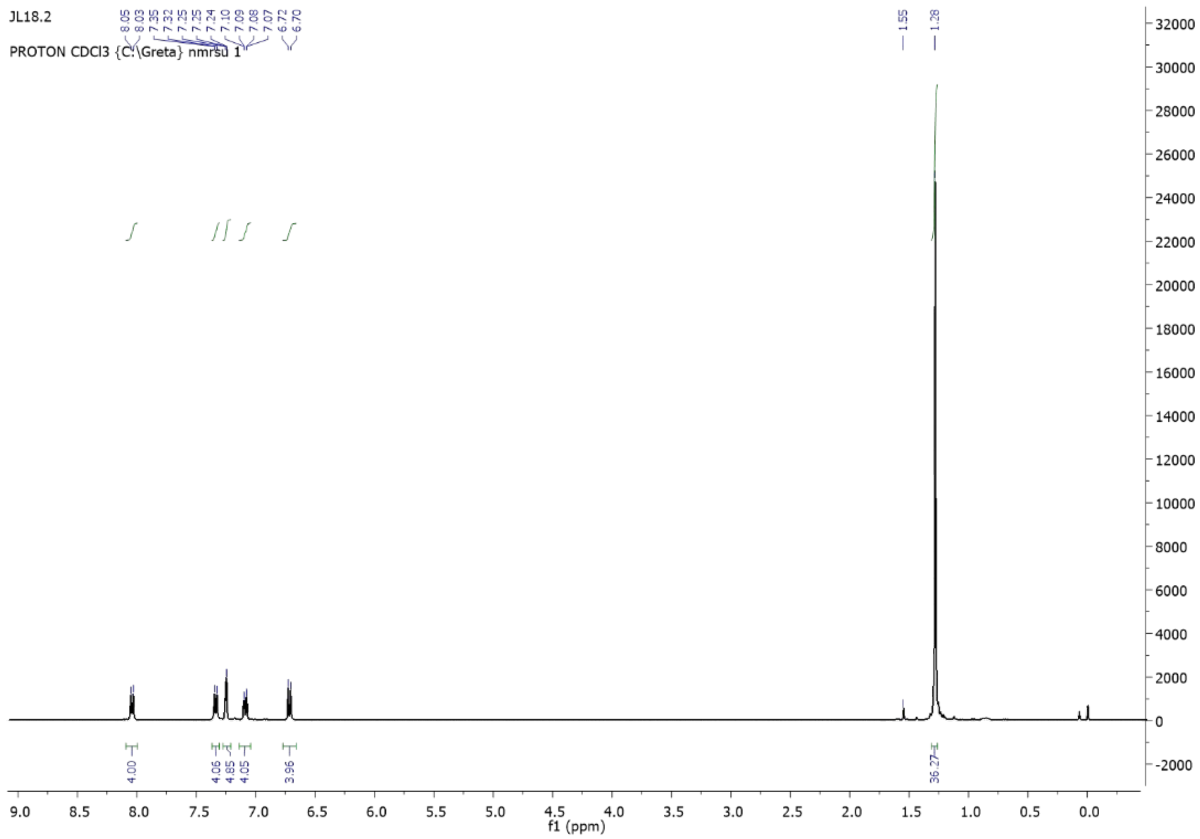

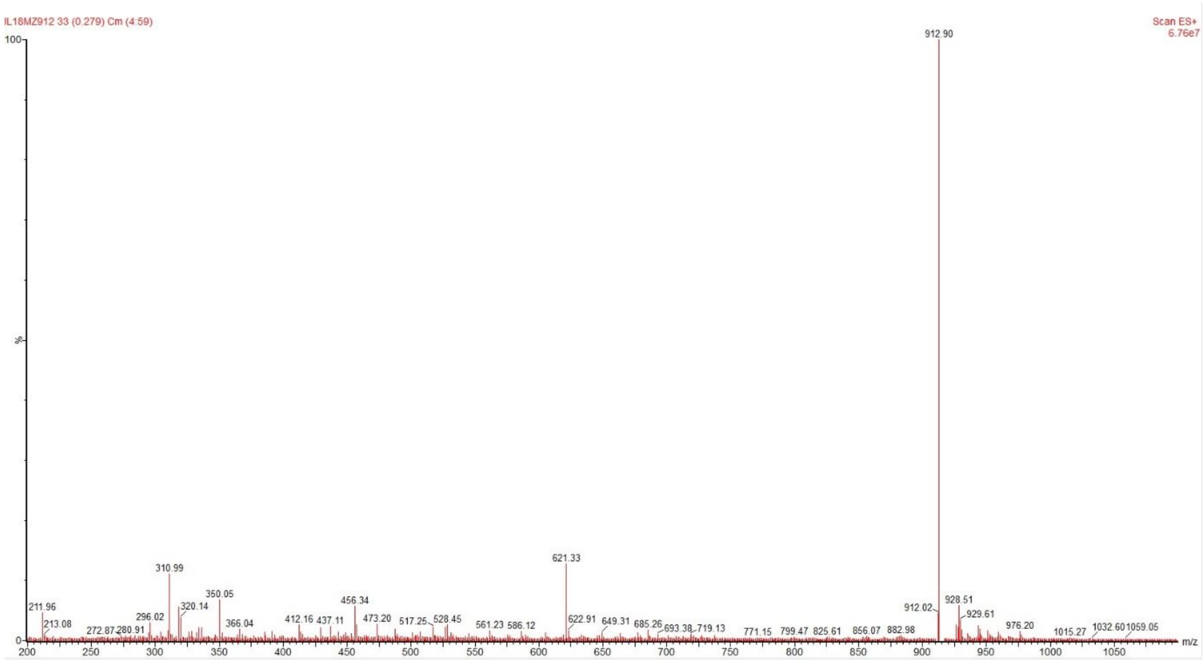

Fig. S3. ^{1}H-NMR and MS spectra of **TTP-PTB**

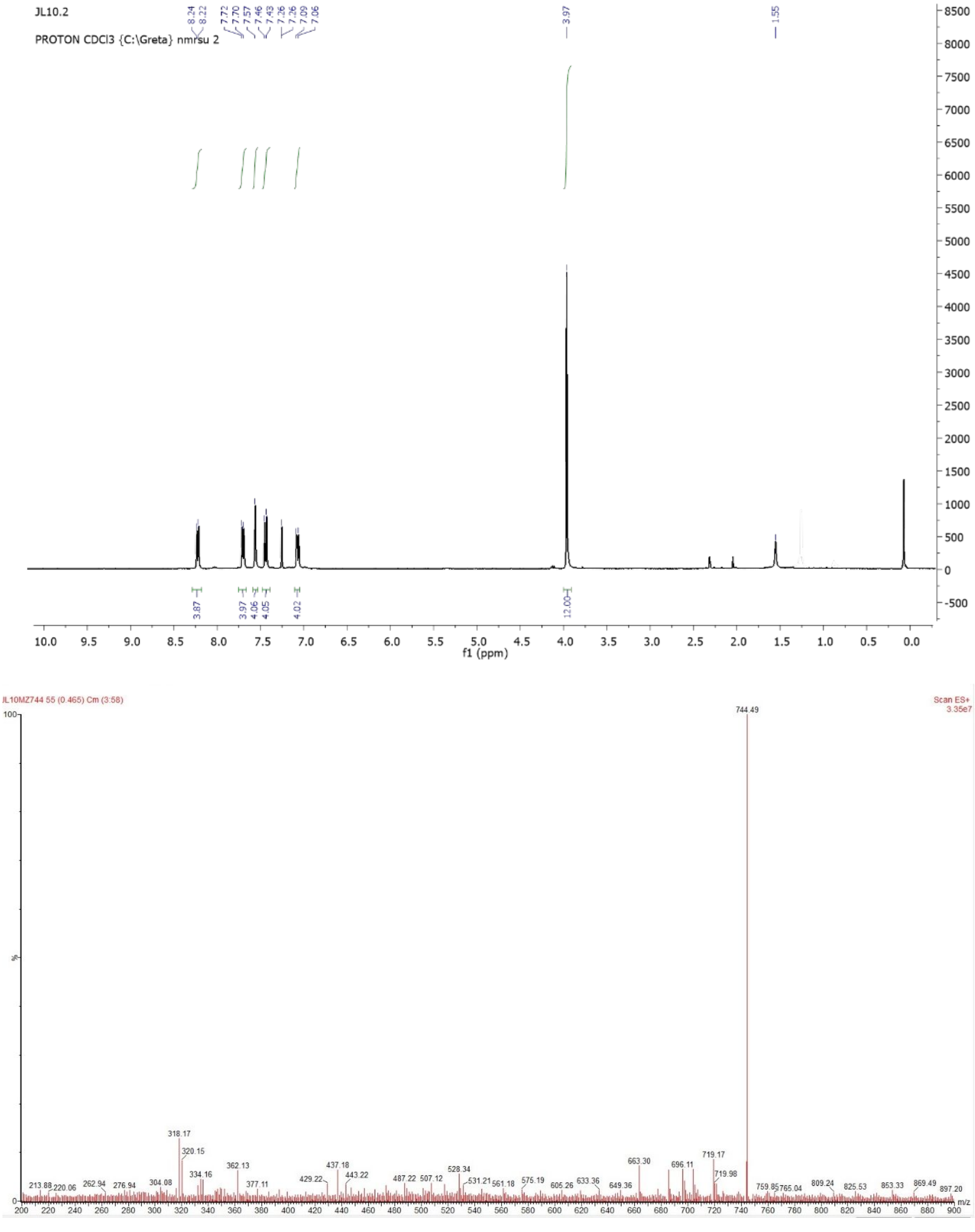

Fig. S4. ^{1}H-NMR and MS spectra of **TTP-CzM**

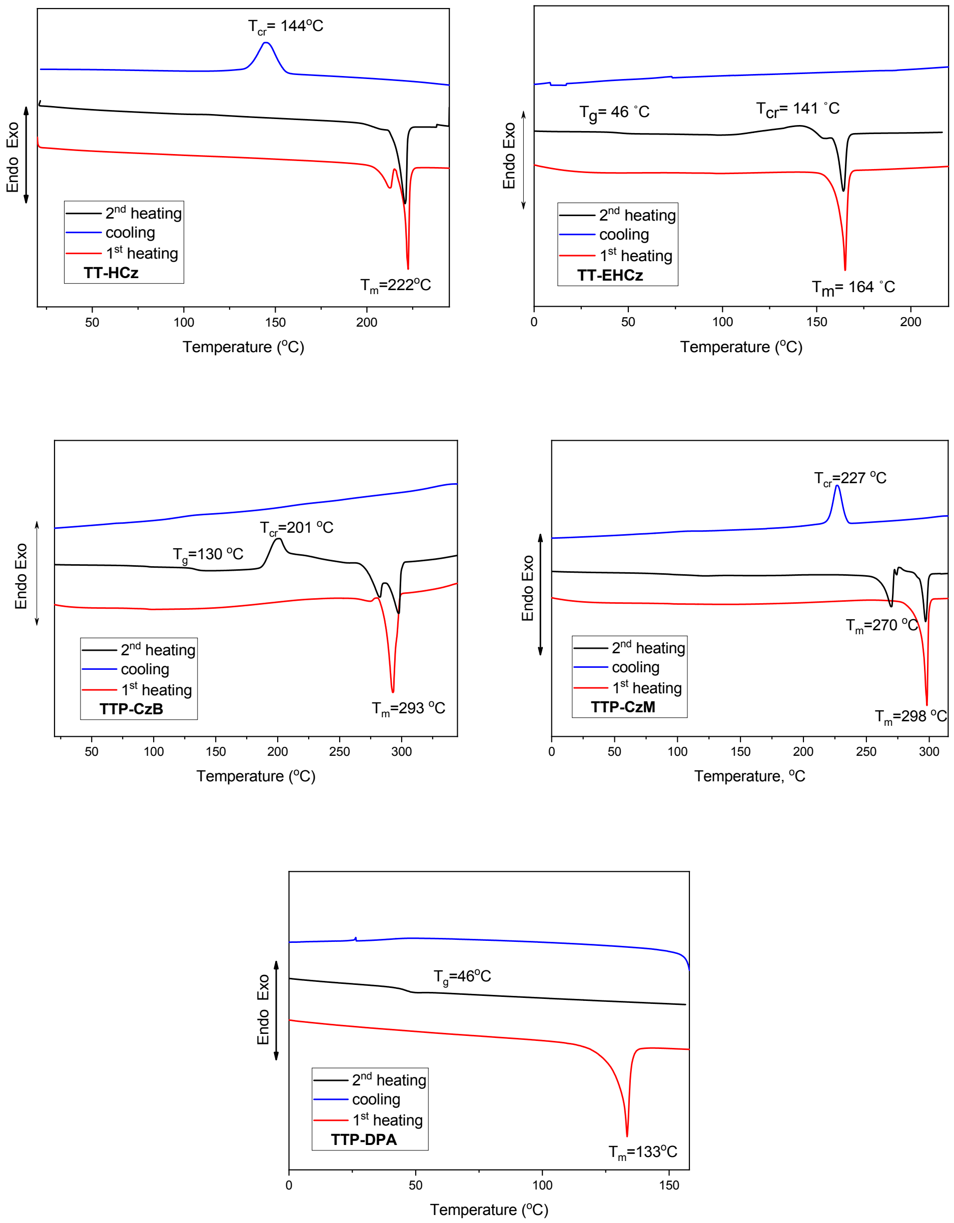

Fig.S5. DSC curves of TT-based compounds recorded at the heating rate of 10 °C min^{-1} in nitrogen atmosphere.

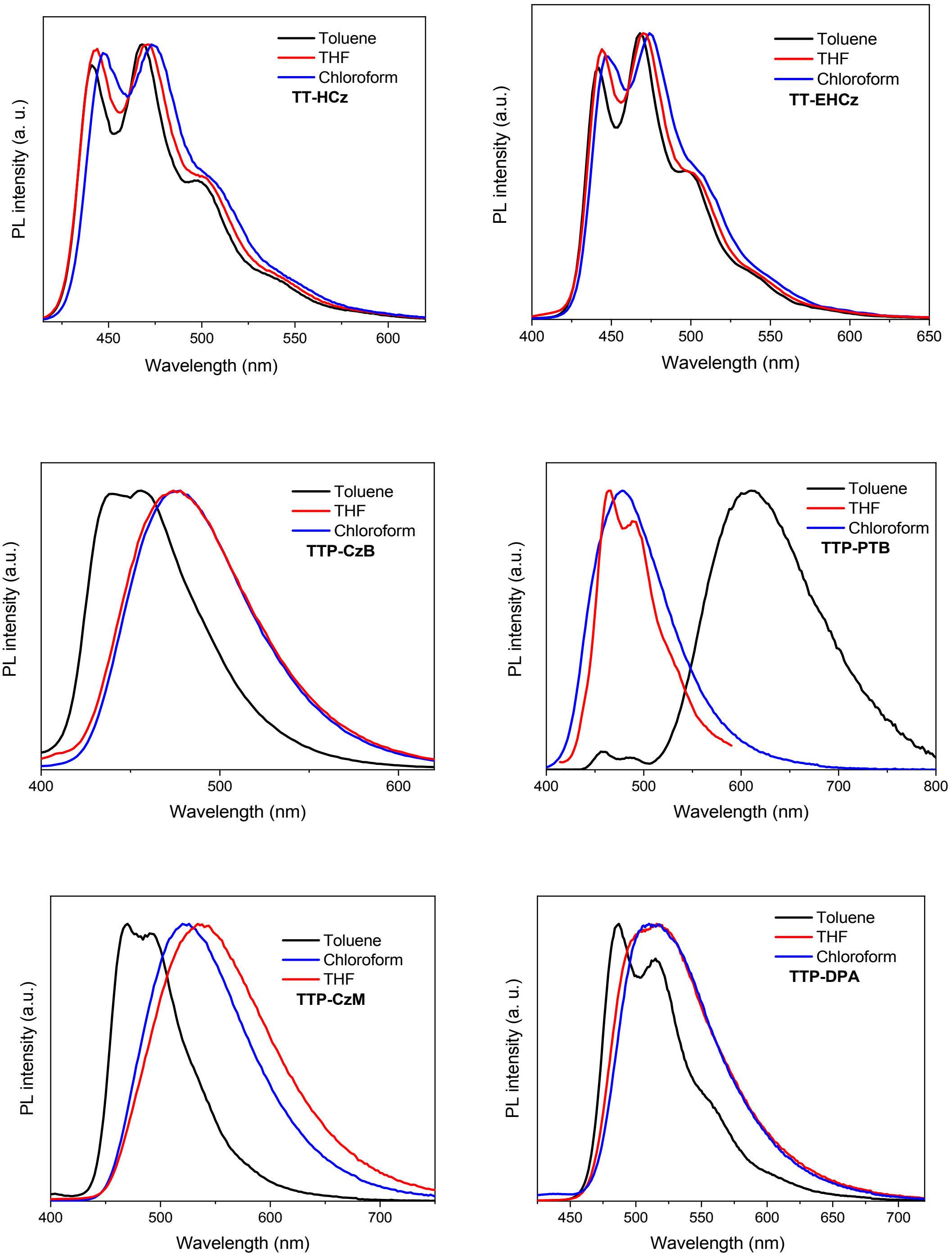

Fig. S6. PL spectra of the dilute toluene, THF and chloroform solutions of TT derivatives.

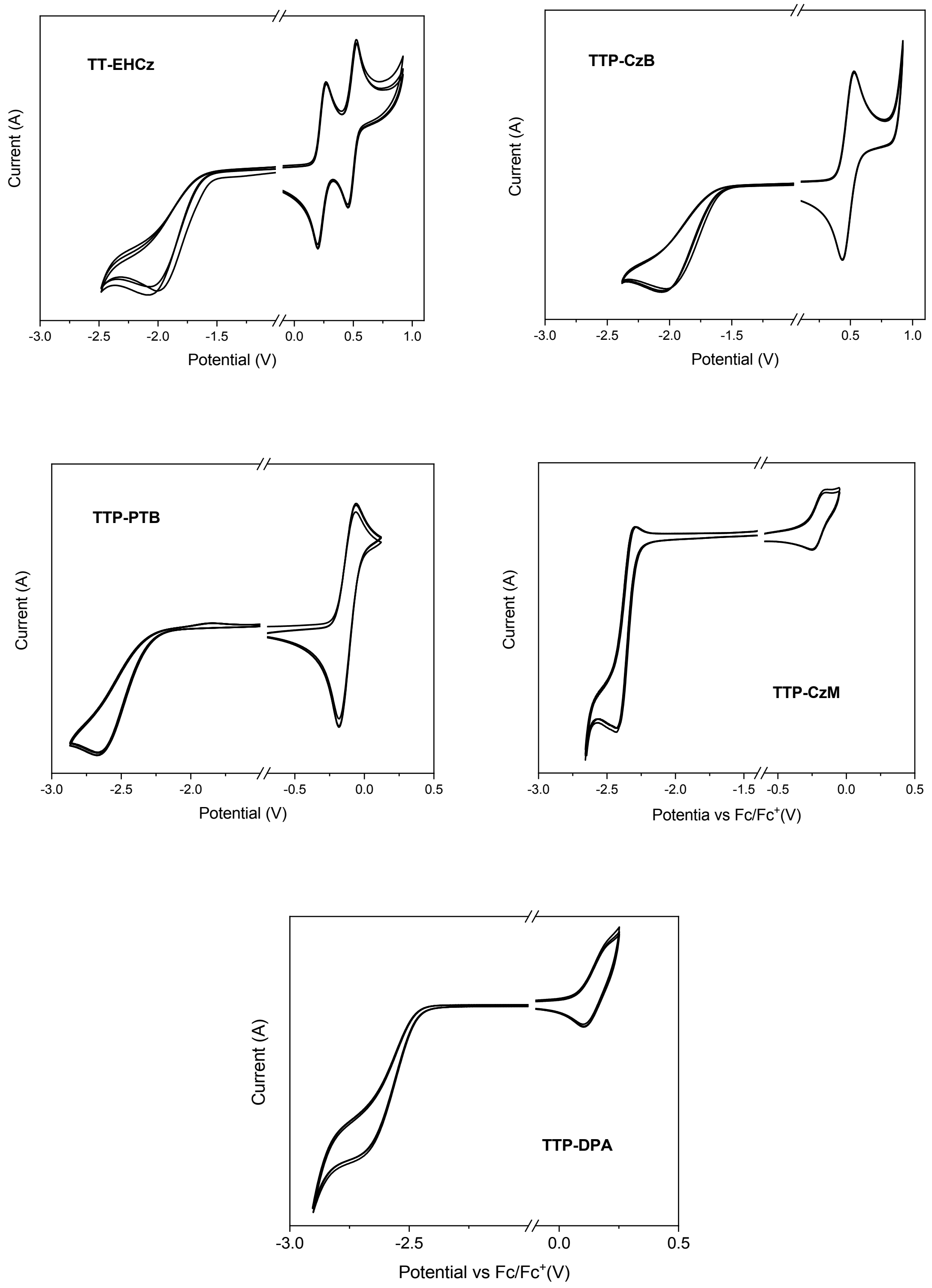

Fig. S7. Cyclic voltammograms of TT derivatives recorded for 0.1 M Bu_4NBF_4/dichloromethane solution at scan rate of 0.1 mVs^{-1}.

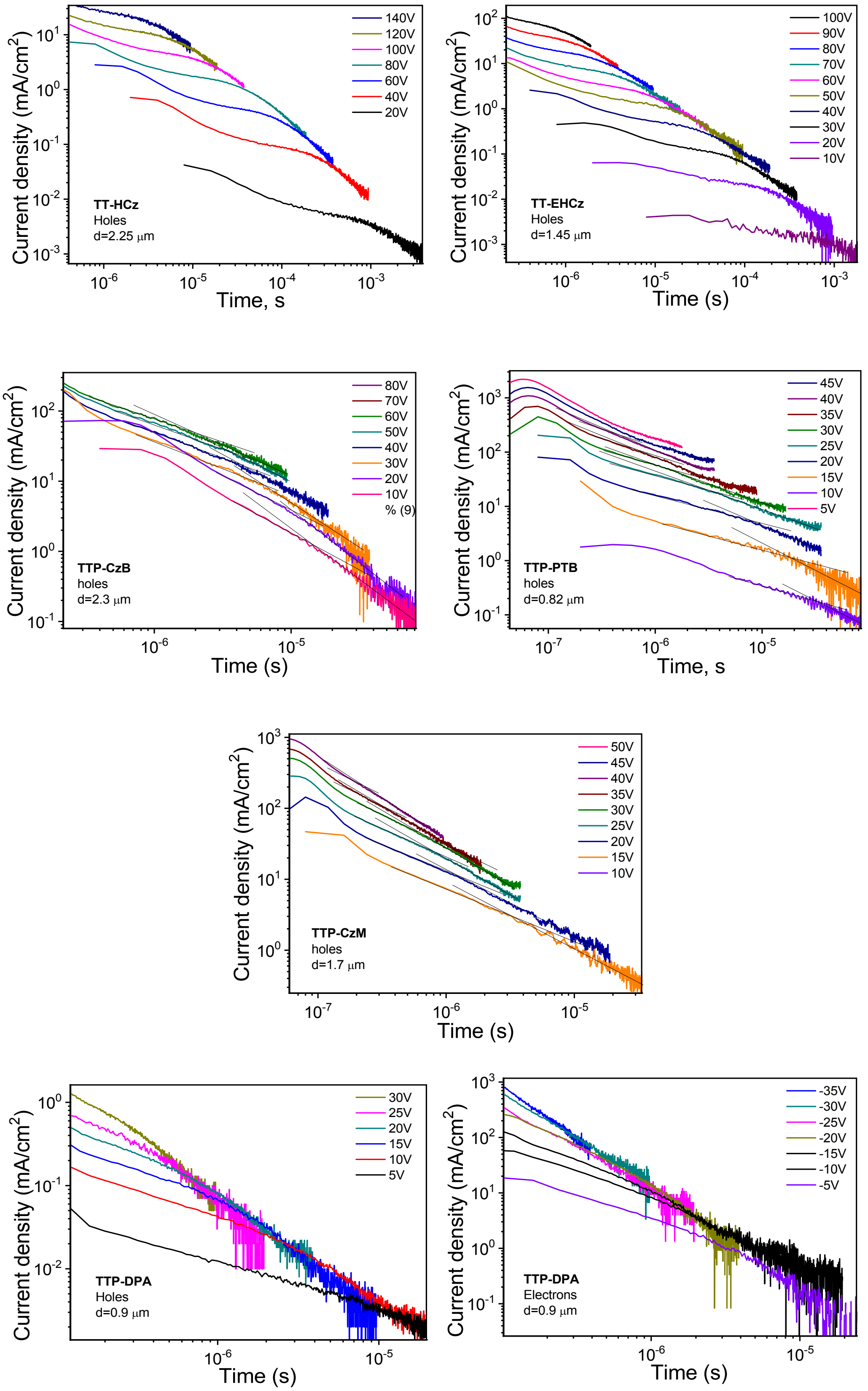

Fig. S8. TOF signals of tested TT-based compounds.

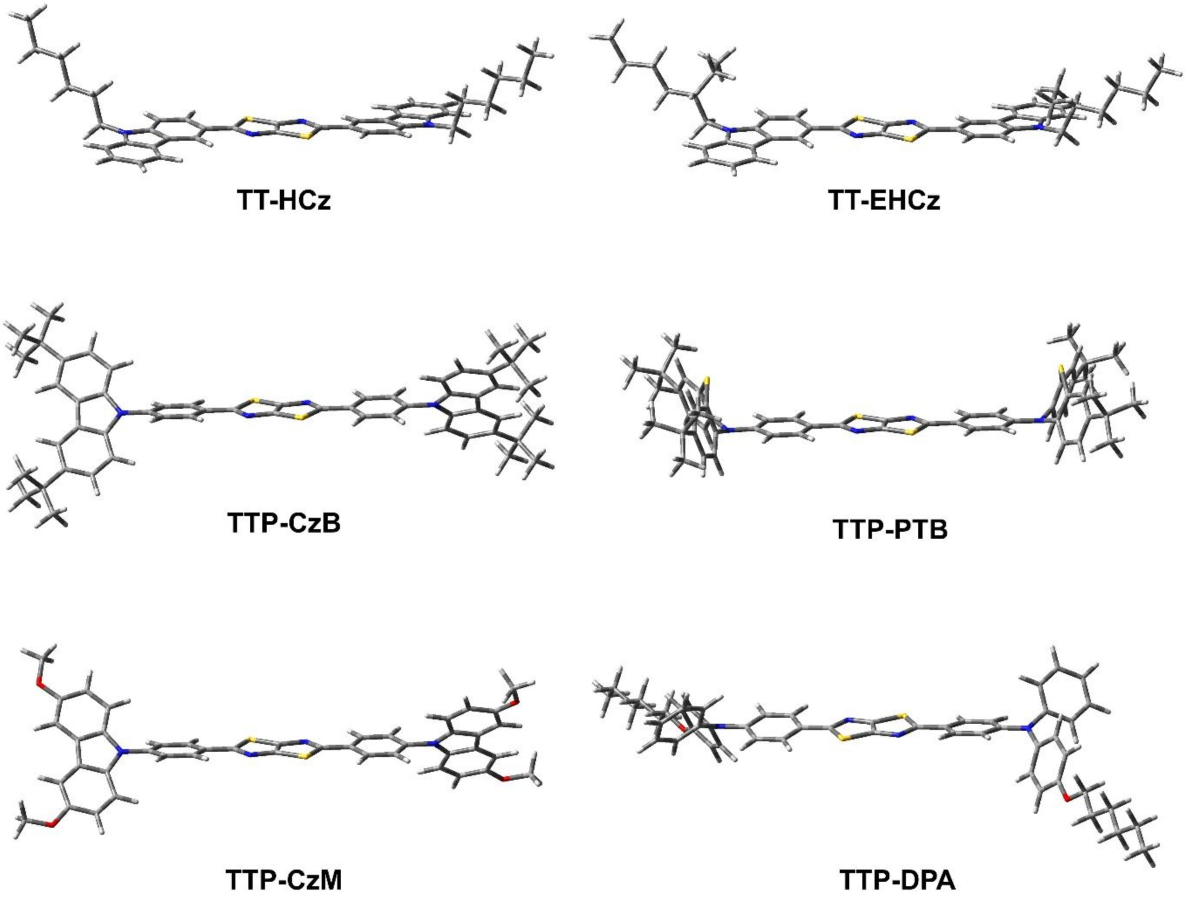

Fig. S9. MN15/6-31+G(d) optimized geometries of considered compounds in gas phase.

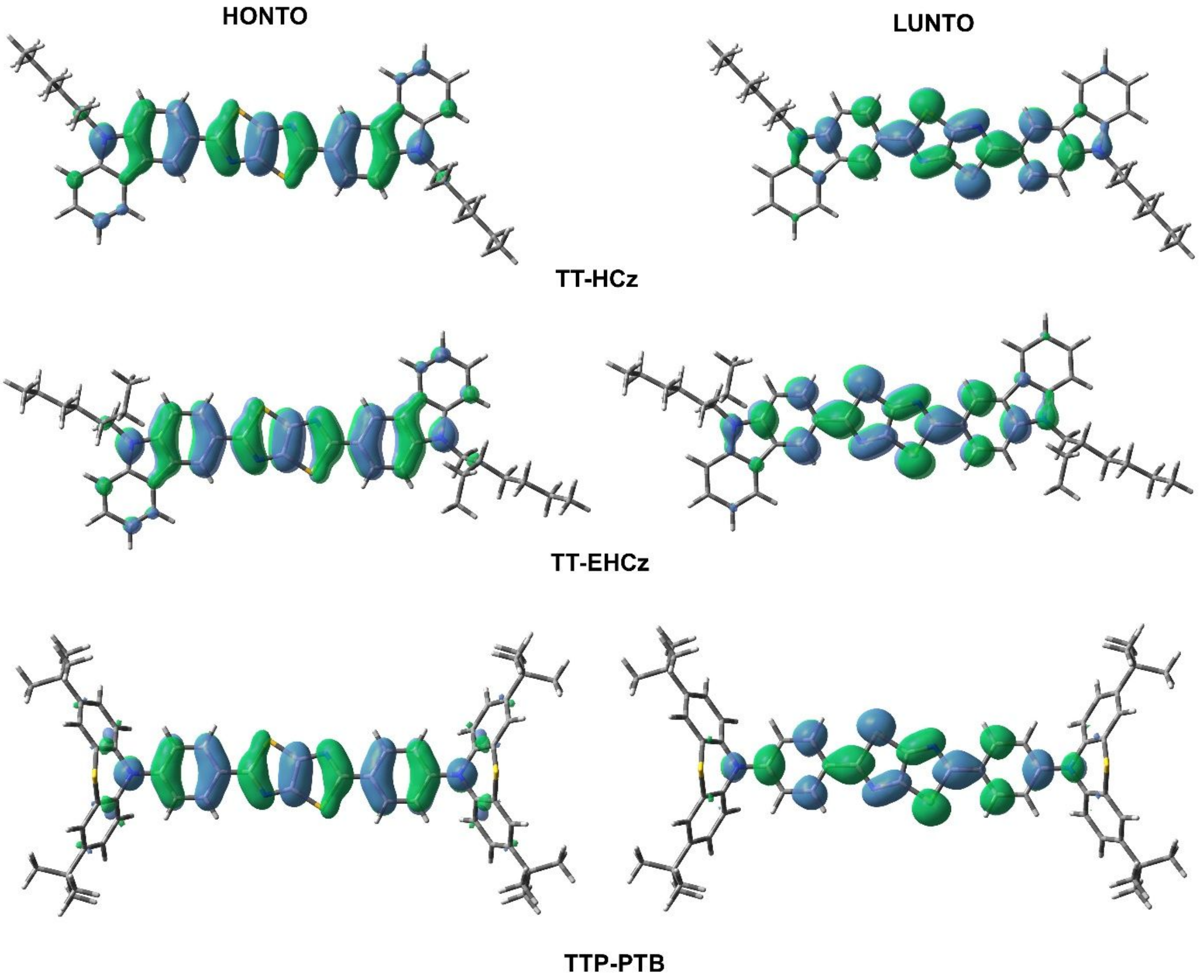

Fig. S10. MN15/6-31+G(d) calculated plots of S1→S0 NTOs for compounds **TT-HCz**, **TT-EHCz**, **TTP-PTB** in THF.

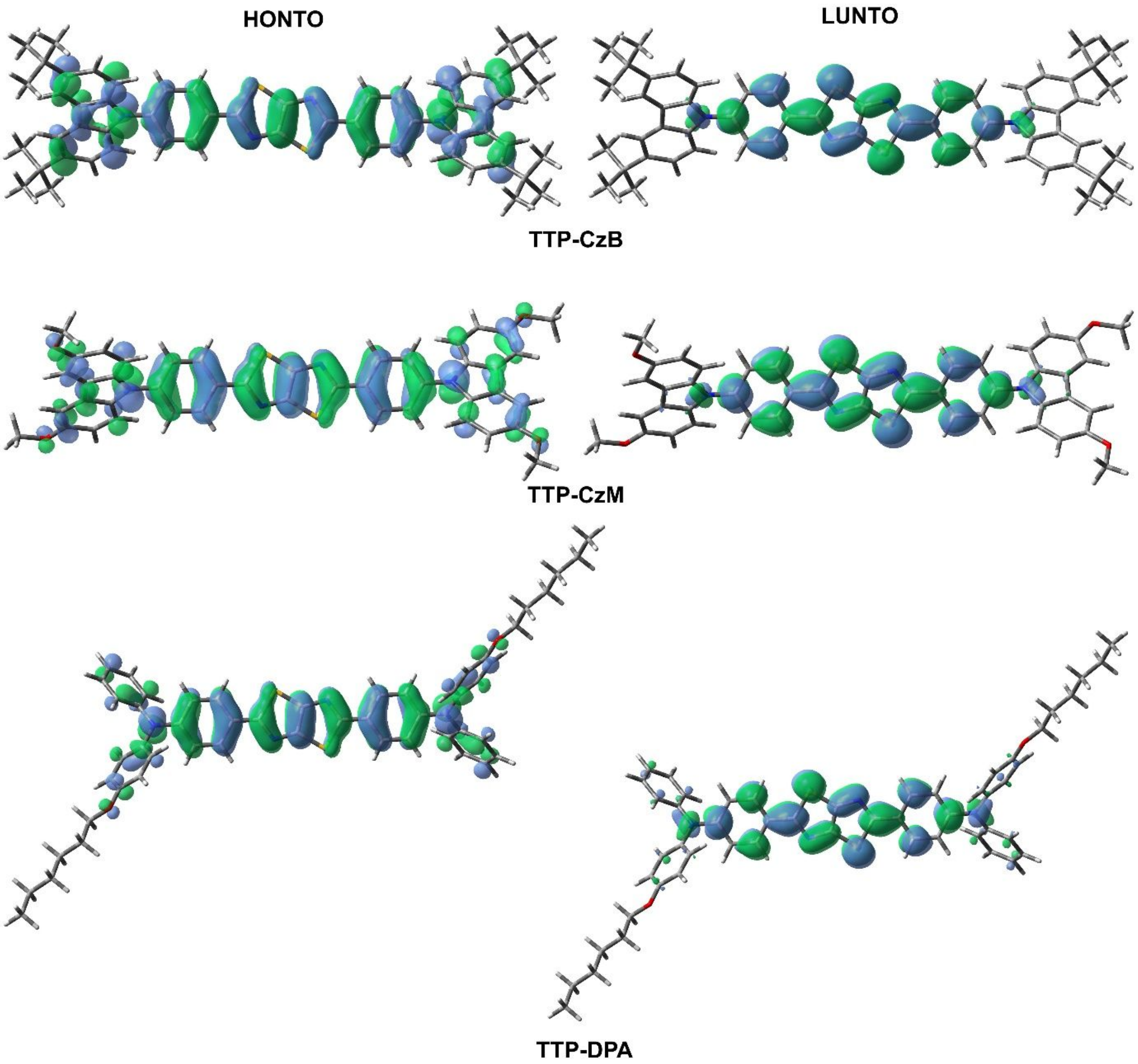

Fig. S11. MN15/6-31+G(d) calculated plots of S1→S0 NTOs for compounds **TTP-CzB**, **TTP-CzM**, **TTP-DPA** in THF.

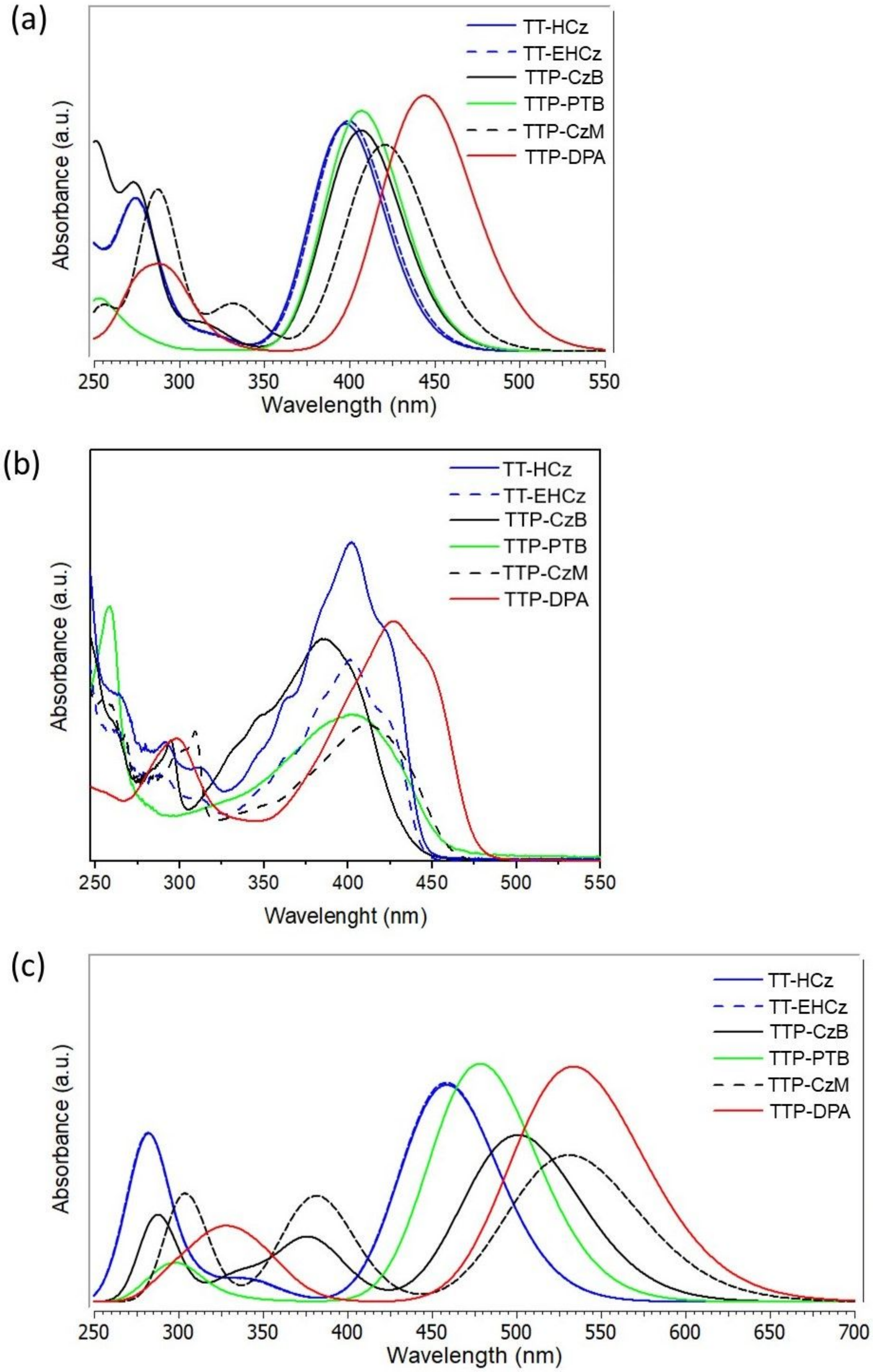

Fig. S12. MN15/6-31+G(d) calculated (a), experimental (b) and B3LYP-GD3BJ/6-31+G(d) [1,2] calculated (c) absorption spectra of TT-based compounds in THF.

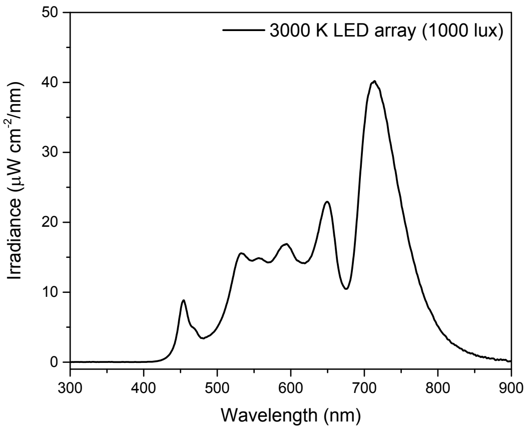

Fig. S13 The emission spectrum of an LED array simulated 3000K LED lamp.

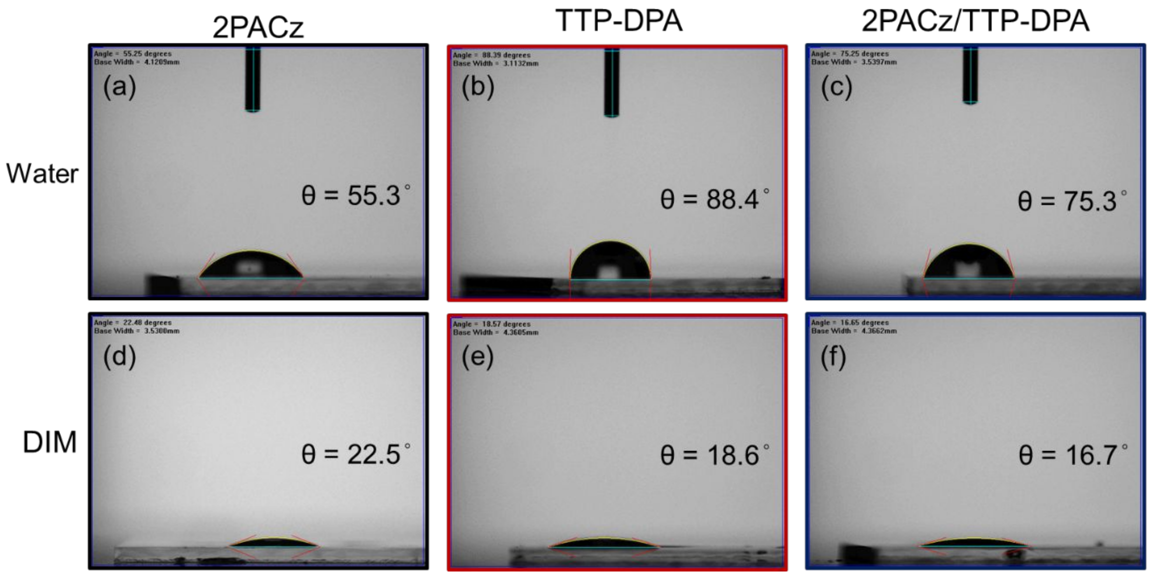

Fig. S14. Water contact angle measurements of 2PACz (a), compound **TTP-DPA** (b) and 2PACz/ **TTP-DPA** (c). DIM contact angle of 2PACz (d), compound **TTP-DPA** (e) and 2PACz/ **TTP-DPA** (f).

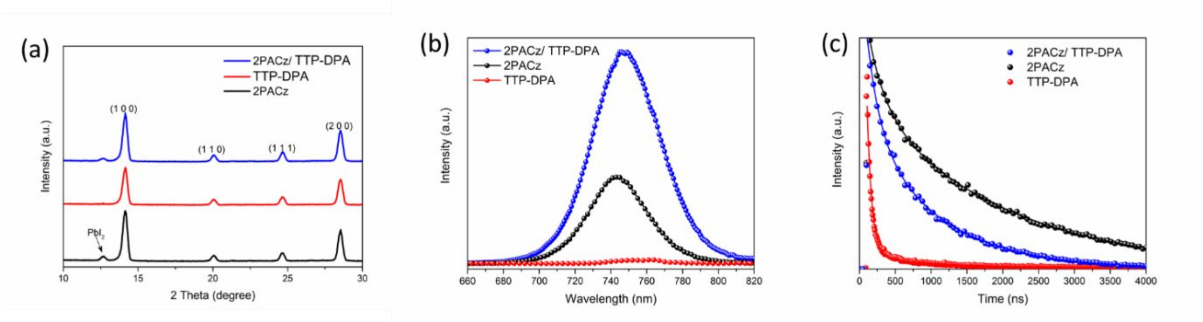

Fig. S15. XRD patterns (a), PL(b) and TCSPC(c) of perovskite deposited on various HSLs.

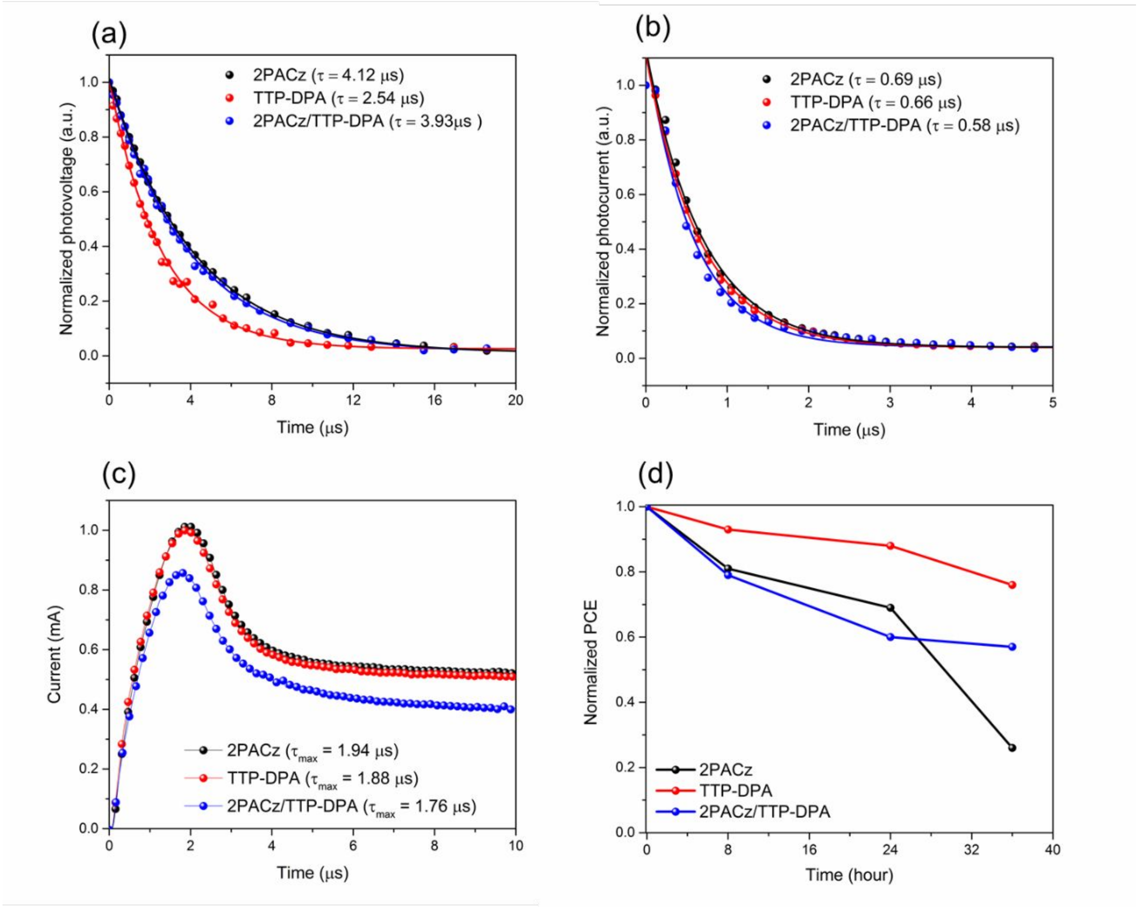

Fig. S16. TPV (a), TPC (b) and Photo-CELIV (c) curves of devices with various HSLs. Thermal stability of the unencapsulated devices (d).

Table S1. B3LYP-GD3BJ/6-31+G(d) calculated wavelengths corresponding to the first absorption maxima (λ^1_{ABS}), wavelengths corresponding to maxima in photoluminescence spectrum (λ_{PL}), dihedral angles between donor and acceptor moieties (α), values of the largest coefficients in the CI expansion (c), oscillator strengths (f), overlaps between functions C_+ and C_- (S_{+-}-index), energy gap between the singlet and triplet states (ΔE_{S-T}) and energies of HOMO and LUMO of TT derivatives in THF.

Parameter	**TT-HCz**	**TT-EHCz**	**TTP-CzB**	**TTP-PTB**	**TTP-CzM**	**TTP-DPA**
λ^1_{ABS} [nm]	459 (402)[a]	459 (401)	501 (386)	479 (403)	531 (413)	533 (427)
α_1 and α_2 [^{0}]	11	12	5 and 51	1 and 90	11 and 49	5 and 29
c(HOMO→LUMO)	0.702	0.702	0.696	0.701	0.696	0.699
f	1.892	1.912	1.456	2.078	1.280	2.052
S_{+-}-index	0.913	0.935	0.899	0.950	0.893	0.909
ΔE_{S-T} [eV] [b]	0.660	0.658	0.508	0.665	0.394	0.514
HOMO [eV]	-5.20	-5.19	-5.30	-5.08	-5.10	-4.89
LUMO [eV]	-1.97	-1.96	-2.36	-1.99	-2.34	-2.09
λ_{PL} [nm]	554 (444, 470)	555 (444, 470)	583 (476)	578 (464, 488)	616 (536)	636 (516)
α_1 and α_2 [^{0}]	0	0	0 and 48	0 and 90	0 and 51	1 and 38
c(HOMO→LUMO)	0.704	0.704	0.701	0.703	0.700	0.702

[a] - The experimental values of λ^1_{ABS} and λ_{PL} are given in brackets, [b] - Energy gap between the singlet and triplet states (ΔE_{S-T}) was calculated as energy difference between S1 and T1 excited states having the ground state geometry in THF.

Table S2. The performance of PSCs with different HSLs and various concentrations of compound **TTP-DPA** under 1 sun.

Concentration of **TTP-DPA** (mg mL^{-1})	Perovskite bandgap (eV)	J_{SC} (mA/cm^2)	V_{OC} (V)	FF (%)	PCE (%)
0.5	1.70	11.3	0.92	68.4	7.6
1.0		15.2	1.01	74.1	11.4
1.5		18.6	1.05	77.5	15.1
3.0		19.8	1.09	79.3	17.1

HSL	Perovskite bandgap (eV)	J_{SC} (mA/cm^2)	V_{OC} (V)	FF (%)	PCE (%)
Free (ITO-only)	1.70	18.6	0.53	43.9	4.4
**TT-HCz**		18.6	0.75	73.9	10.3
**TTP-PTB**	1.77	13.0	0.93	78.9	9.6
**TTP-CzM**		8.1	0.30	69.7	1.7

Table S3. Device performance of the PSCs, including PSCs and iPSCs.

Illumination: AM 1.5G 100 mW cm^{-2}

HSL	J_{SC} (mA cm^{-2})	V_{OC} (V)	FF (%)	PCE (%)	PCE_{Best} (%)
2PACz	19.4 ± 1.1	1.18 ± 0.02	74.5 ± 2.4	17.0 ± 0.5	17.4
**TTP-DPA**	19.1 ± 0.7	1.08 ± 0.06	78.4 ± 3.9	16.0 ±0.7	17.1
2PACz/ **TTP-DPA**	19.6 ± 0.2	1.18 ± 0.02	79.2 ± 1.5	18.4 ± 0.7	19.1

Illumination: 3000K LED 1000 lux

HSL	P_{in} (μWcm^{-2})	P_{out} (μWcm^{-2})	J_{SC} (μA cm^{-2})	V_{OC} (V)	FF (%)	PCE (%)	PCE_{Best} (%)

2PACz		181.8 ± 7.1	274.9 ± 13.0	0.92 ± 0.05	72.2 ± 3.9	30.6 ± 1.2	32.2
**TTP-DPA**	596.0	143.4 ± 10.3	252.9 ± 16.6	0.77 ± 0.06	74.0 ± 4.9	24.1 ± 1.8	26.8
2PACz/ **TTP-DPA**		200.6 ± 12.8	262.1 ± 11.4	1.00 ± 0.05	76.3 ± 2.5	33.7 ± 2.2	37.0

Table S4. Contact angles and surface energies of 2PACz, **TTP-DPA** and 2PACz/ **TTP-DPA**.

Film	θ_{water} (°)	θ_{DIM} (°)	γ_p (mN m^{-1})	γ_d (mN m^{-1})	γ_{total} (mN m^{-1})
2PACz	53.1	22.5	19.8	47.1	66.9
**TTP-DPA**	87.9	18.5	4.2	48.2	52.4
2PACz/ **TTP-DPA**	75.2	15.3	9.2	49.0	58.2

References

(1) Grimme, S.; Ehrlich, S.; Goerigk, L. Effect of the Damping Function in Dispersion Corrected Density Functional Theory. *J Comput Chem* **2011**, *32* (7), 1456–1465. https://doi.org/10.1002/jcc.21759.

(2) Becke, A. D. Density-Functional Thermochemistry. III. The Role of Exact Exchange. *J Chem Phys* **1993**, *98* (7), 5648–5652. https://doi.org/10.1063/1.464913.
